# Low-molecular-weight anti-HIV-1 agents targeting HIV-1 capsid proteins†

**DOI:** 10.1039/d2ra06837k

**Published:** 2023-01-12

**Authors:** Takuya Kobayakawa, Masaru Yokoyama, Kohei Tsuji, Masayuki Fujino, Masaki Kurakami, Takato Onishi, Sayaka Boku, Takahiro Ishii, Yutaro Miura, Kouki Shinohara, Yuki Kishihara, Nami Ohashi, Osamu Kotani, Tsutomu Murakami, Hironori Sato, Hirokazu Tamamura

**Affiliations:** a Institute of Biomaterials and Bioengineering, Tokyo Medical and Dental University (TMDU) 2-3-10 Kandasurugadai, Chiyoda-ku Tokyo 101-0062 Japan tamamura.mr@tmd.ac.jp; b Pathogen Genomics Center, National Institute of Infectious Diseases Musashimurayama 208-0011 Tokyo Japan hirosato@nih.go.jp; c AIDS Research Center, National Institute of Infectious Diseases Shinjuku-ku Tokyo 162-8640 Japan tmura@nih.go.jp; d Showa Pharmaceutical University Machida 194-8543 Tokyo Japan

## Abstract

The HIV-1 capsid is a shell that encapsulates viral RNA, and forms a conical structure by assembling oligomers of capsid (CA) proteins. Since the CA proteins are highly conserved among many strains of HIV-1, the inhibition of the CA function could be an appropriate goal for suppression of HIV-1 replication, but to date, no drug targeting CA has been developed. Hydrophobic interactions between two CA molecules through Trp184 and Met185 in the protein are known to be indispensable for conformational stabilization of the CA multimer. In our previous study, a small molecule designed by *in silico* screening as a dipeptide mimic of Trp184 and Met185 in the interaction site was synthesized and found to have significant anti-HIV-1 activity. In the present study, molecules with different scaffolds based on a dipeptide mimic of Trp184 and Met185 have been designed and synthesized. Their significant anti-HIV activity and their advantages compared to the previous compounds were examined. The present results should be useful in the design of novel CA-targeting anti-HIV agents.

## Abstract 2

Design and synthesis of MKN-3 derivatives: a new class of small molecules, MKN-3 derivatives, were developed based on *in silico* screening, as dipeptide mimics of Trp184 and Met185 at the hydrophobic interaction site between two capsid (CA) proteins.

## Graphical abstract

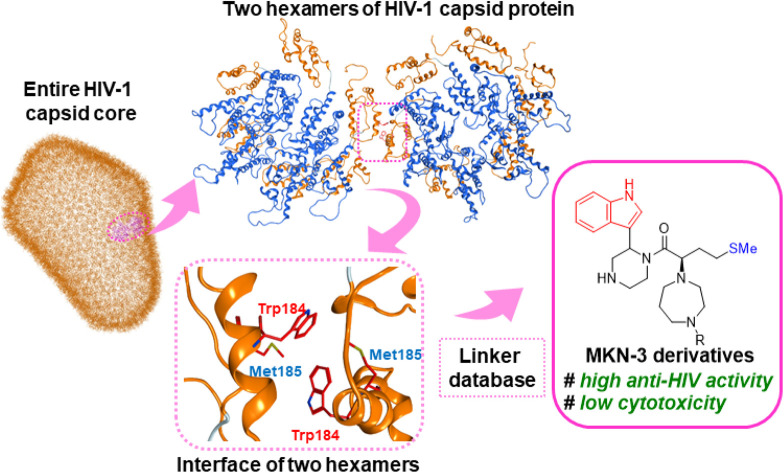

## Introduction

The pandemic of the novel COVID-19, which is caused by a positive-strand RNA virus SARS-CoV-2, continues in 2022. Overcoming such infectious diseases has always been a major task for human beings.^1^ Human immunodeficiency virus type 1 (HIV-1) is classified as a member of the retrovirus family, and HIV-1 infects CD4-positive macrophage or T-cells, finally causing acquired immunodeficiency syndrome (AIDS). To date, many anti-HIV-1 drugs^2–4^ beginning with inhibitors of three major viral enzymes, reverse transcriptase,^5^ protease^6^ and integrase,^7,8^ have been developed, and the use of these drugs in combination antiretroviral therapy (cART) has brought great success to the chemotherapy of HIV infectious diseases.^2–4^ Some drugs are picked up in Table 1. There have been serious shortcomings however, including the emergence of mutant viral strains with multi-drug resistance, the appearance of significant side effects and the cost of the resulting drugs. In a persistent effort to resolve these problems and increase the alternatives and repertoires of anti-HIV-1 drugs, we have sought anti-HIV agents with various mechanisms of action such as co-receptor CXCR4 antagonists,^9–15^ CD4 mimics,^16–21^ fusion inhibitors,^22–25^ integrase inhibitors^26–28^ and inhibitors of viral uncoating and viral assembly.^29–33^ Among these targets, inhibitors of viral uncoating and viral assembly^29–33^ are the focus of this paper. The HIV-1 Gag precursor protein Pr55Gag produces the capsid (CA) protein, which is composed of N- and C-terminal domains (NTD/CTD) and is highly conserved among numerous HIV strains.^34,35^ Many CA protein molecules are structurally assembled by oligomerization of hexamers and pentamers,^36^ and form a CA core with a conical structure,^37,38^ which encloses the RNA genome, the reverse transcriptase and the integrase. In addition, Pr55Gag produces the matrix (MA) protein, which contributes to the assembly of the virion shell.^39,40^ The MA and CA proteins are thought to be valid targets for inhibition of viral replication. Consequently, several MA and CA fragment derivatives were found to have anti-HIV activity in our^29–33^ and other laboratories.^41–44^ These peptide-derived inhibitors must penetrate cell membranes to inhibit the viral uncoating and assembly because these replication steps are based on the MA/CA degradation and oligomerization and are performed inside host cells. For this purpose, an octa-arginyl group^45^ was added into the earlier peptide-derived inhibitors, described above, to enhance their cell membrane permeability.^29–33^ Since small molecules might themselves have cell membrane permeability without an octa-arginyl group, it is desirable to search for small compounds with inhibitory activity against viral uncoating and assembly. Although several small compounds have been discovered to date,^46–64^ none has progressed to clinical trials with the exception of lenacapavir, formerly GS-6207.^59^

**Table 1:** Some reported anti-HIV drugs

Compound	Structure	Function	IC_50_/EC_50_	Reference
			(IC_90_/EC_90_)	
Maraviroc	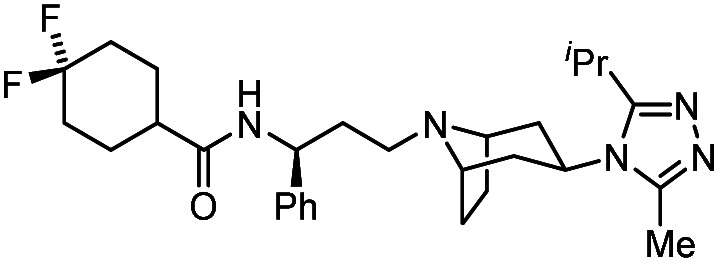	CCR5 antagonist	(1.0 nM)	65
Enfuvirtide (T-20)	Peptide	Fusion inhibitor	3.0 nM	66
Fostemsavir	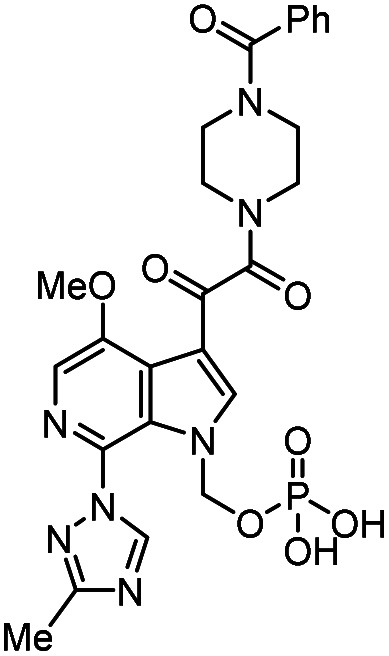	Entry inhibitor	0.7 nM	67
Ibalizumab	Antibody	CD4 binding inhibitor	0.29 nM	68
GSK3640254	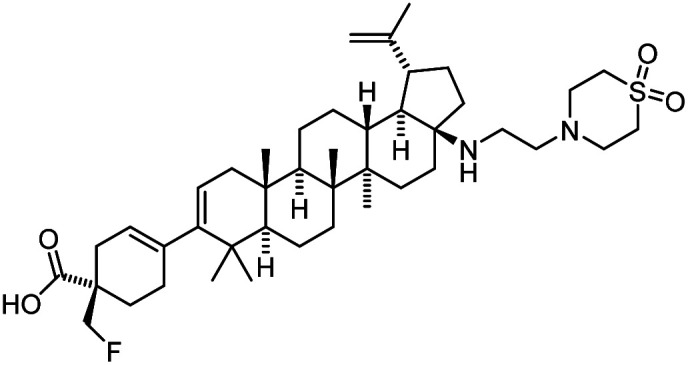	Maturation inhibitor	(33 nM)	69
Cabotegravir	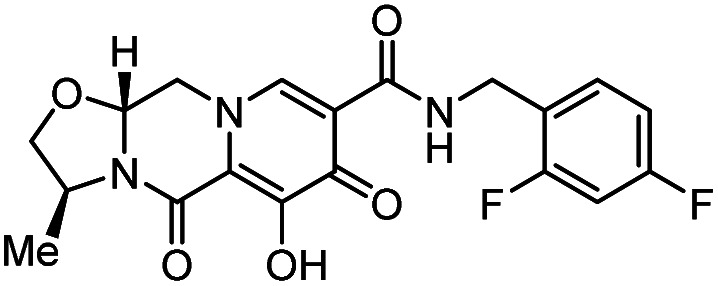	Integrase inhibitor	0.25 nM	70
Lenacapavir	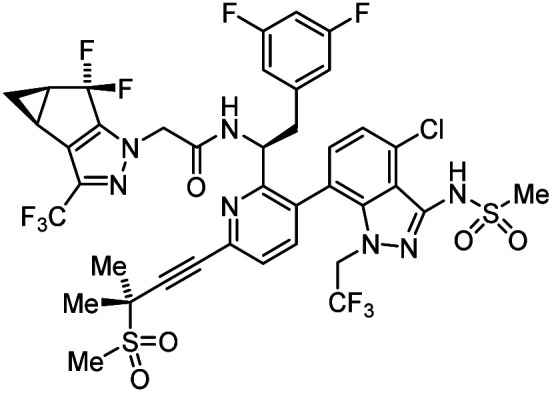	Capsid inhibitor	100 pM	71

**Fig. 1:**
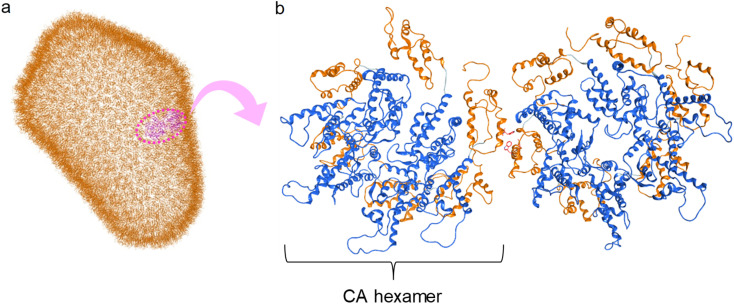
(a) Capsid corn structure (PDB ID: 3J3Q); (b) interaction between two hexamers of CA proteins (blue: N-terminal domain (NTD), orange: C-terminal domain (CTD)).

The structural analysis of CA proteins has revealed a hydrophobic interaction through Trp184 in Helix 9 of CTD of one CA molecule and Met185 of the other molecule.^36–38,72^ This interaction is indispensable for conformational stabilization of the CA multimer to form the CA core (Fig. 1). Viral mutants with Trp184Ala and Met185Ala mutations have very weak dimeric interactions between two CA molecules resulting in an abnormal morphology of the viral particles, and consequently causing no infectivity.^73^ The Trp184-Met185 sequence is highly conserved among natural HIV/simian immunodeficiency virus (SIV) strains.^20^ In addition, our previous CA-derived fragment peptide, which includes Trp184 and Met185, has significant anti-HIV activity.^74^

**Fig. 2:**
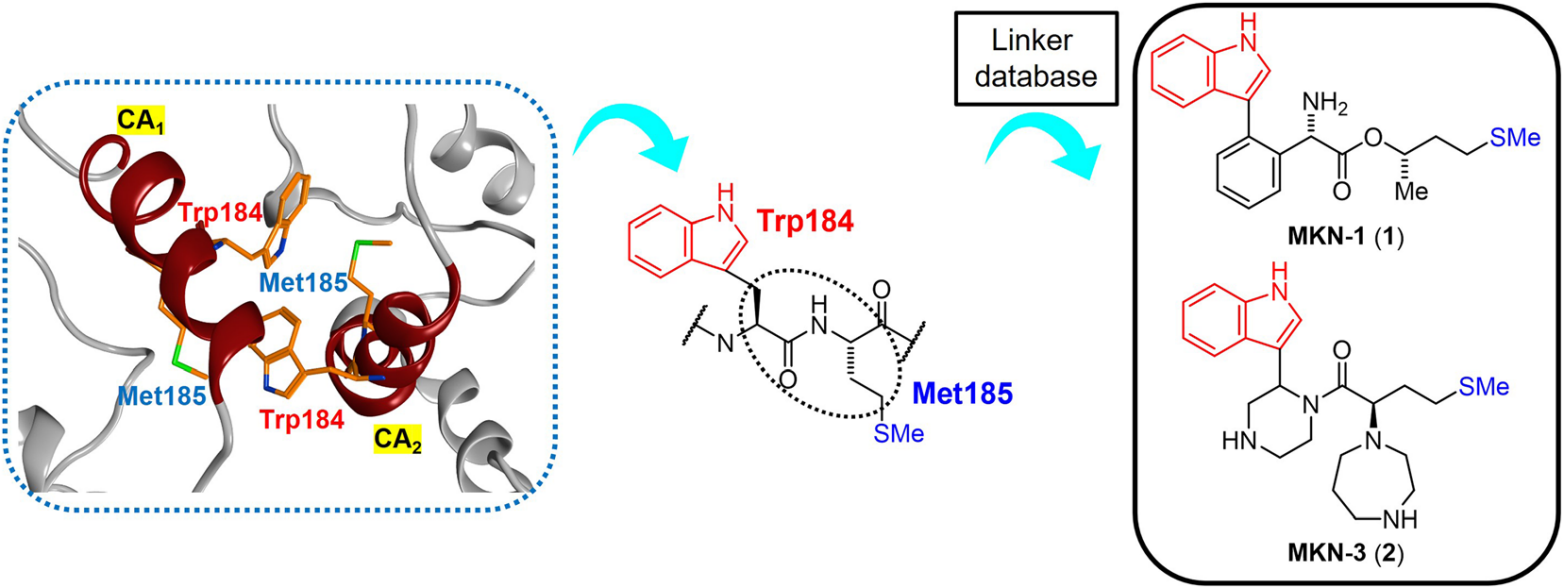
The hydrophobic interaction between two CA molecules *via* Trp184 and Met185 (PDB ID: 3J34, orange: carbon atom, blue: nitrogen atom, green: sulfur atom) (left), extraction of the Trp (W) 184 - Met (M) 185 sequence from the CA_2_ molecule (center) and the design of dipeptide mimics of Trp184 and Met185 screened using the linker database to provide MKN-1 (1) and MKN-3 (2) (right). CA_1_ and CA_2_: two CA molecules.

The site of this hydrophobic interaction through Trp184 of one molecule and Met185 of a second molecule might be considered to be a valid target of small drugs for CA dysfunction. Our strategy has involved design of small molecules, which might bind to the above site, using *in silico* screening.^75^ Briefly, a series of dipeptide mimics of Trp184 and Met185 were designed using the Molecular Operating Environment (MOE) (Chemical Computing Group Inc., Montreal, Quebec, Canada). The structure of one monomer molecule of the CA protein dimer (PDB ID: 3J34) remained as a receptor, and the backbone structure of Trp184 and Met185 of the other monomer molecule was removed. The side-chain structures of these two residues were left in place, and the compounds affecting the backbone structures crosslinking two side-chain functional groups were screened using the linker database provided by the Scaffold Replacement application of MOE to bind to the above receptor side (Fig. 2).

Screened dipeptide mimic candidates were selected using the following scores which involve binding affinity for receptors, ligand efficacy (London dG), topological polar surface area, molecular weight, log of the octanol/water partition coefficient (*S* log *P*), and an estimate of the feasibility of synthetic access.^76^ London dG values show binding affinities of available compounds for target proteins; smaller values mean higher binding affinities, and compounds with London dG values of <−6 have higher binding affinity for a CA molecule when compared to the interaction between two CA molecules because the London dG value of the CA dimer is approximately −6. This screening strategy provides some candidates with useful binding affinity, including MKN-1 (1) with a London dG value of −9.134, which had been found previously to have significant anti-HIV activity.^75^ This screening identified another candidate MKN-3 (2) with London dG value of −9.555, signifying a higher binding affinity for a CA molecule than that of MKN-1 (1). In the current study, MKN-3 (2) and several of its derivatives were synthesized, and their anti-HIV activity and cytotoxicity were evaluated.

## Results and discussion

### Retrosynthetic analysis for the synthesis of MKN-3 (2)

**Scheme 1:**
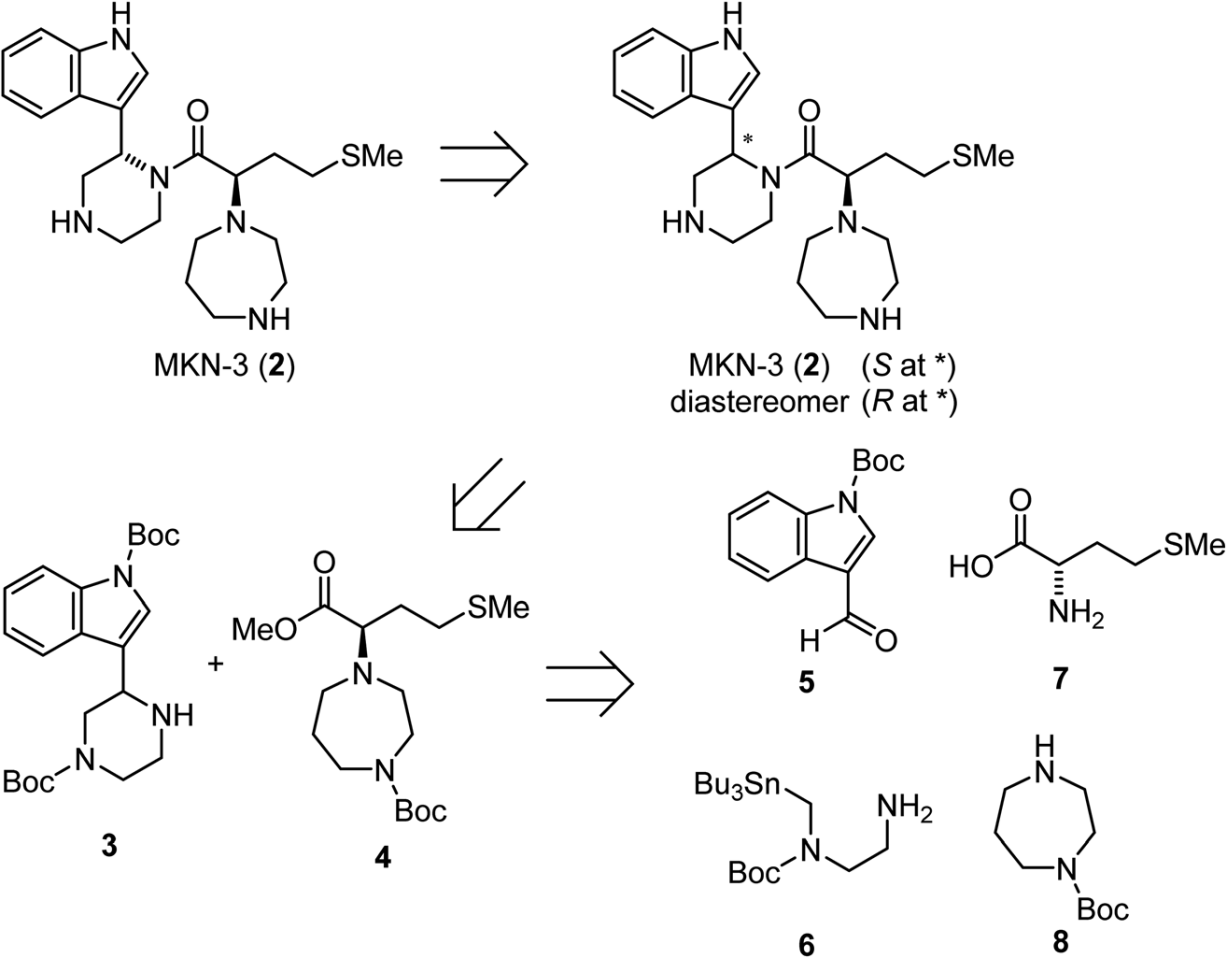
Retrosynthetic analysis of MKN-3 (2).

A possible synthesis of MKN-3 (2) accompanied by its diastereomer was outlined. For its construction, MKN-3 (2) with two chiral centers was divided into four segments 5,^77^6,^78^7 and 8 based on a retrosynthetic analysis (Scheme 1).

### Synthesis of MKN-3 (2)

**Scheme 2:**
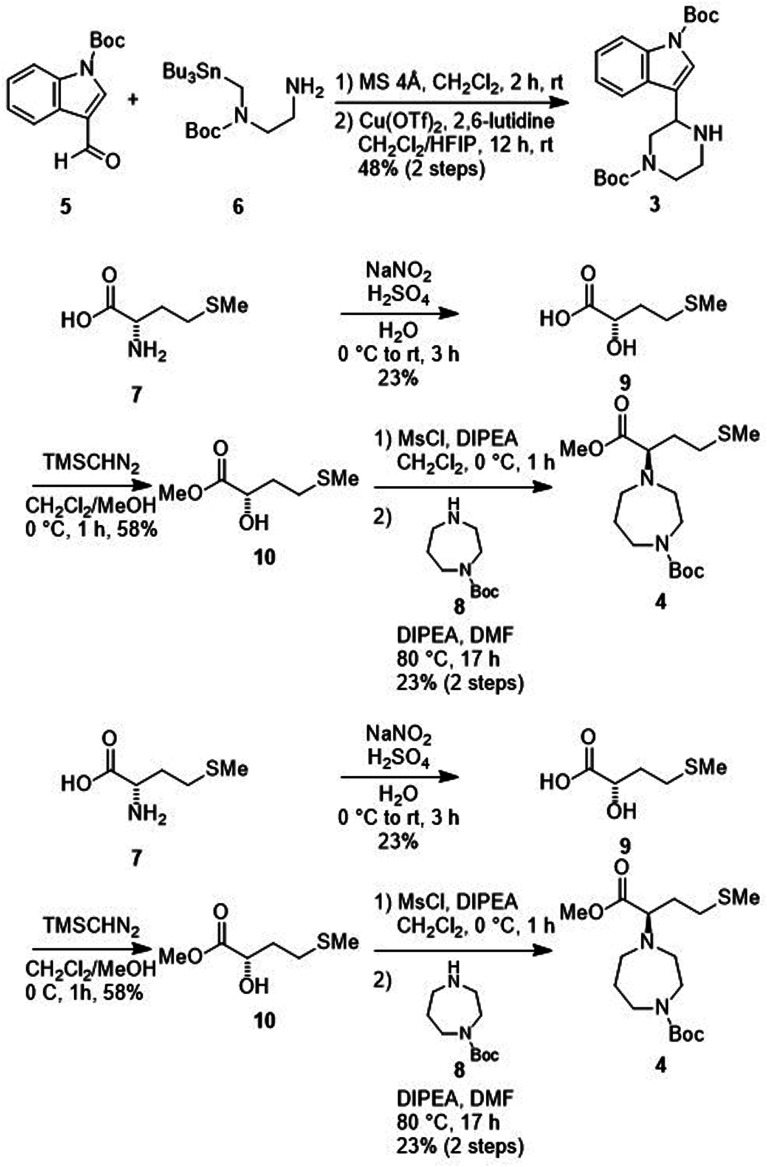
Synthesis of MKN-3 (2) (diastereomixture). ^#^HPLC purification using 0.1% (v/v) trifluoroacetic acid (TFA) containing eluents after the reaction.

Treatment of the aldehyde (5)^77^ with the SnAP Pip reagent (6)^78^ gave the corresponding imine and was followed by the subsequent 6-endo cyclization to yield an amine (3) (Scheme 2). Reaction of the α-hydroxycarboxylic acid (9), which was prepared from l-methionine (7),^79^ with trimethylsilyl (TMS) diazomethane produced the ester (10). Mesylation of 10 and a subsequent S_N_2 reaction with 1-Boc-homopiperazine (8) yielded an ester (4). Saponification of the ester (4), and subsequent condensation with the amine (3) followed by deprotection of two *N*-Boc groups gave MKN-3 (2) and its diastereomer.

### Synthesis of derivatives of MKN-3 (2)

**Scheme 3:**
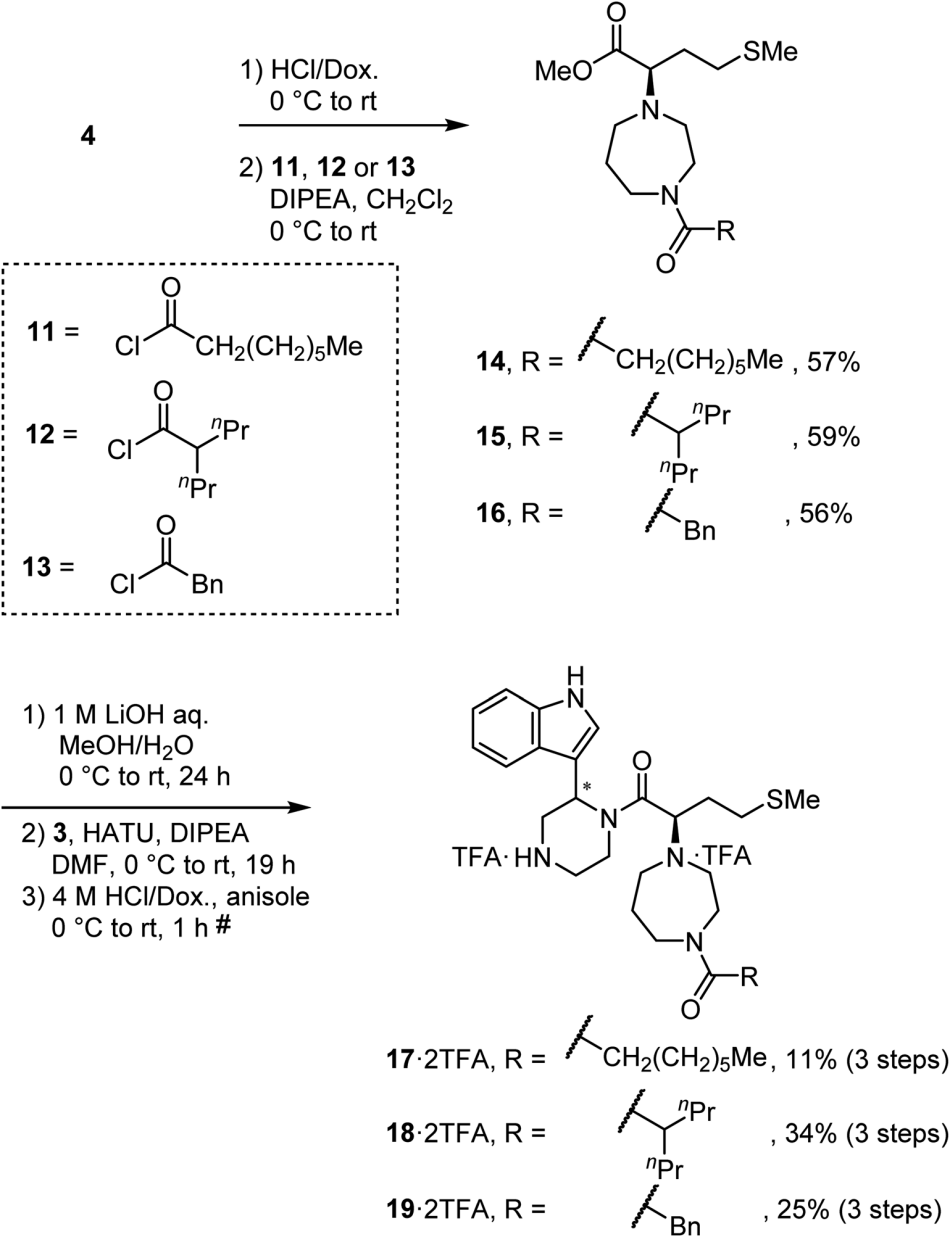
Synthesis of compounds 17, 18 and 19 (diastereomixtures). ^#^HPLC purification using 0.1% (v/v) TFA containing eluents after the reaction.

To synthesize derivatives of MKN-3 (2), some acyl or alkyl groups were introduced after deprotection of the *N*-Boc group of the ester (4) was performed. Treatment with *n*-octanoyl chloride, 2-propylvaleryl chloride or phenylacetyl chloride gave amides 14, 15 and 16, respectively, and this was followed by saponification, condensation with the amine (3) and deprotection of two *N*-Boc groups to yield the MKN-3 derivatives (17, 18 and 19), respectively, as their diastereomixtures (Scheme 3). *In silico* docking simulation was used to validate preservation of binding activity of the MKN-3 derivatives. The MKN-3 derivatives had better binding score London dG values than MKN-1 had (London dG value = −5.90 to −6.50 for MKN-3 derivatives and −5.80 for MKN-1, respectively). The MKN-3 derivatives somehow exhibited variation in the lengths of substituents, suggesting toleration in the size of moiety that does not participate directly to the binding to the CA protein.

**Scheme 4:**
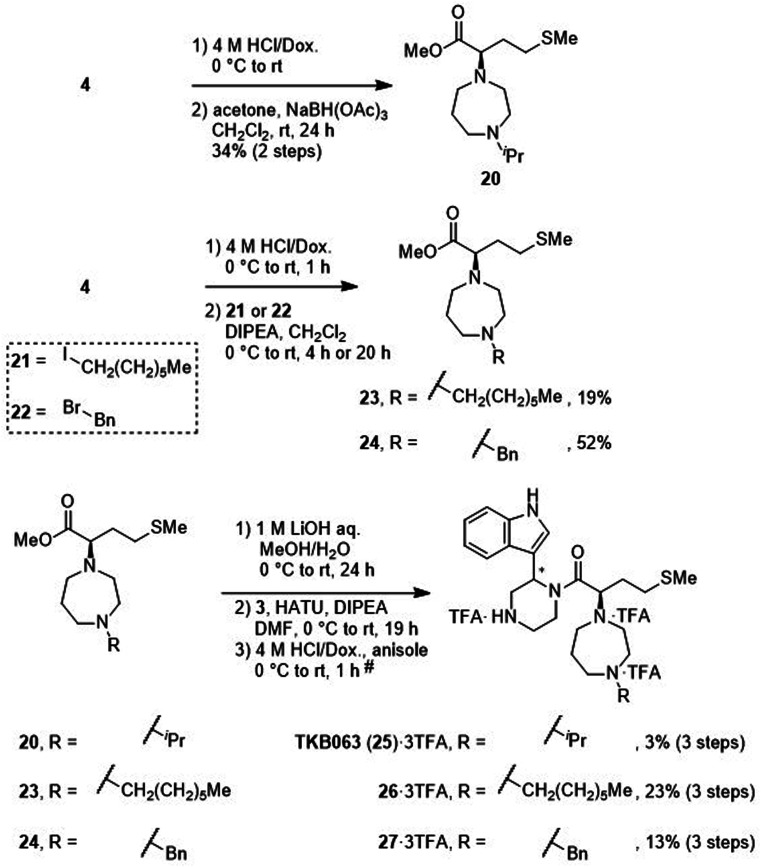
Synthesis of compounds TKB063 (25), 26 and 27 (diastereomixtures). ^#^HPLC purification using 0.1% (v/v) TFA containing eluents after the reaction.

After removal of the *N*-Boc group of the ester (4), treatment with acetone and sodium triacetoxyborohydride gave an amine (20) by reductive amination (Scheme 4). Treatment with 1-iodoheptane (21) or benzyl bromide (22) yielded amines 23 and 24, respectively, *via* an S_N_2 reaction. After saponification of amines 20, 23 and 24, condensation with the amine (3) and deprotection of two *N*-Boc groups gave derivatives TKB063 (25), 26 and 27, respectively, as diastereomixtures.

**Scheme 5:**
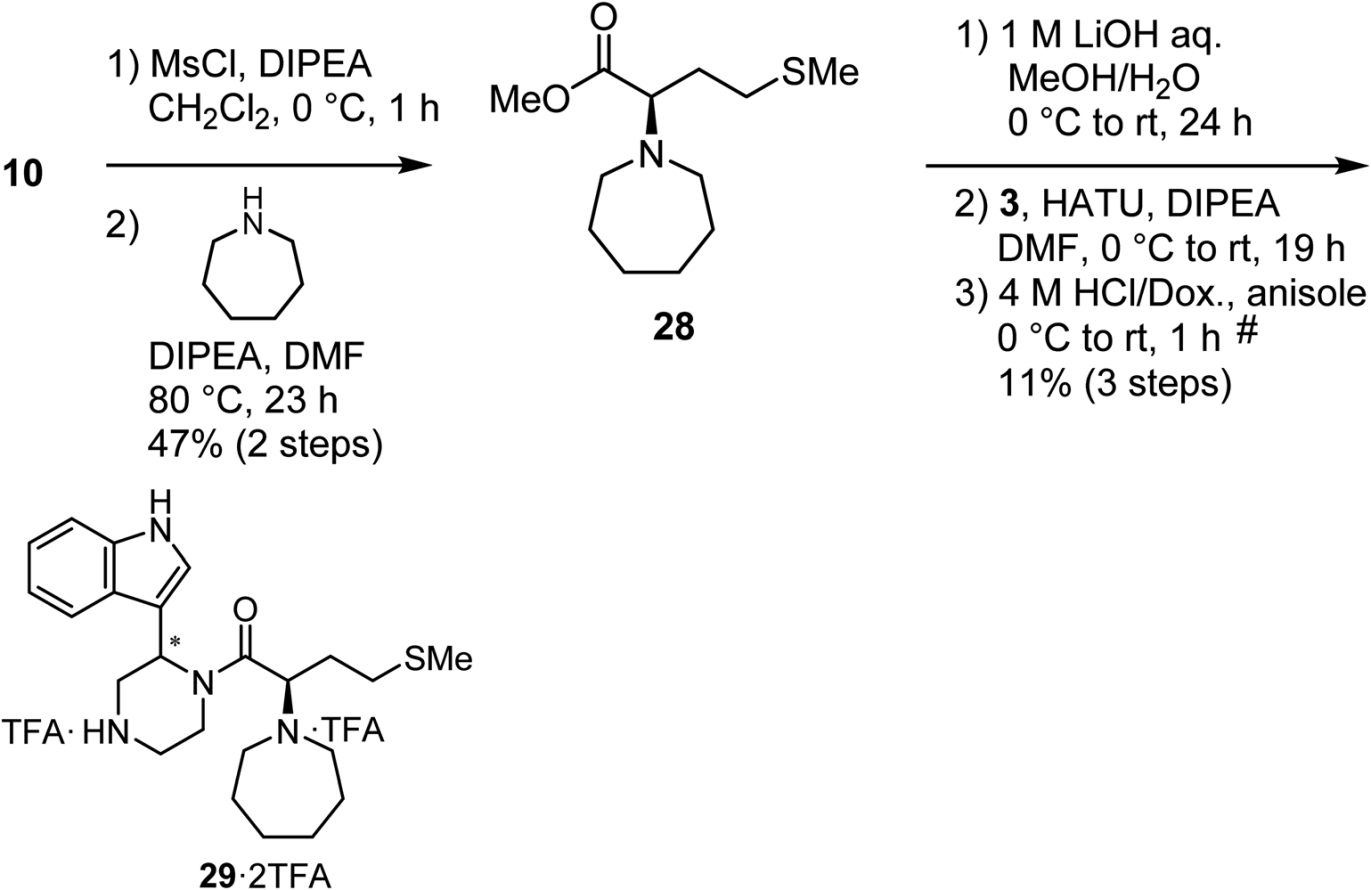
Synthesis of compound 29 (diastereomixture). ^#^HPLC purification using 0.1% (v/v) TFA containing eluents after the reaction.

Mesylation of 10 and a subsequent S_N_2 reaction with hexamethyleneimine yielded an ester (28), and this was followed by saponification, condensation with the amine (3) and deprotection of two *N*-Boc groups to yield the derivative (29) as a diastereomixture (Scheme 5).

**Scheme 6:**
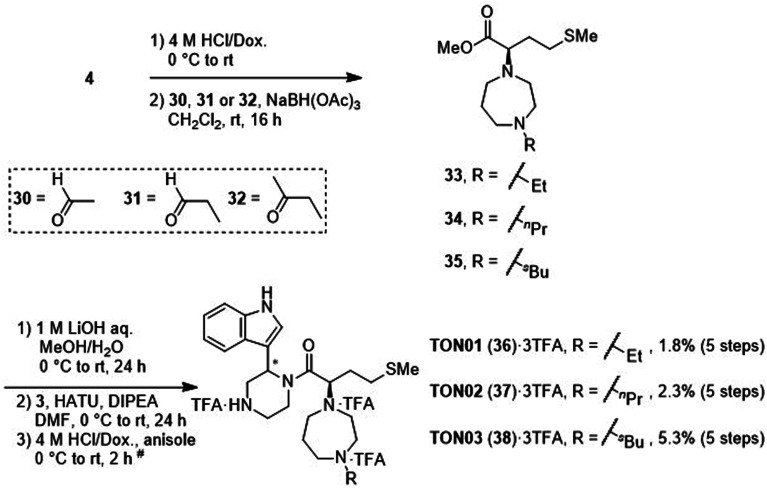
Synthesis of TON01 (36), TON02 (37) and TON03 (38) (diastereomixtures). ^#^HPLC purification using 0.1% (v/v) TFA containing eluents after the reaction.

After deprotection of the *N*-Boc group of the ester (4), treatment with acetaldehyde (30), propionaldehyde (31) or 2-butanone (32) in the presence of sodium triacetoxyborohydride gave amines 33, 34 and 35, respectively, *via* reductive amination (Scheme 6). The synthesis of 20 from 4 is shown in Scheme 4. After saponification of the amine (33, 34 or 35), condensation with the amine (3) and deprotection of two *N*-Boc groups gave derivatives TON01 (36), TON02 (37) and TON03 (38), respectively, as diastereomixtures.

### Evaluation of anti-HIV activity and cytotoxicity

**Fig. 3:**
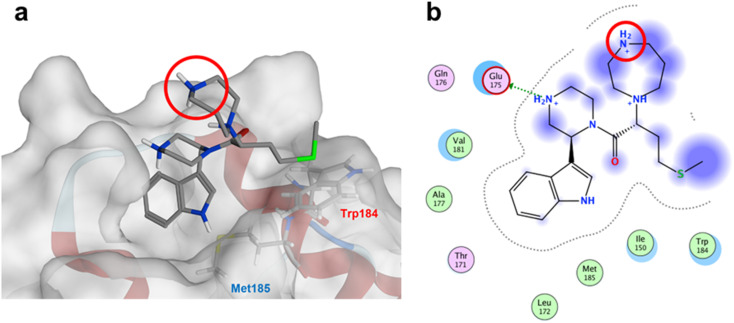
(a) The docking model of MKN-3 (2) and a CA protein (PDB ID: 3J34), blue: nitrogen atom, red: oxygen atom, green: sulfur atom; (b) the docking simulation operated by Molecular Operating Environment (MOE), 2019.01.^66^ Amino acid residues surrounding MKN-3 (2) are shown. Indigo-colored cloud: ligand exposure, light blue shade: receptor exposure. The H_2_N^+^ moiety in the homopiperazine ring is marked by a red circle.

The anti-HIV activity of the synthesized compounds was assessed based on their protection against HIV-1 (NL4-3 strain)-induced cytopathogenicity in MT-4 cells using an MTT assay.^29–33^ The cytotoxicity of these compounds was determined based on reduction of the viability of MT-4 cells, as determined by an MTT assay. These results are shown in Table 2. First, MKN-3 (2), evaluated as the mixture of its diastereomers, showed low anti-HIV activity below 50 μM, but failed to show cytotoxicity below 50 μM. MKN-3 (2) was designed by *in silico* screening in the same way as MKN-1 (1), and in terms of ligand efficacy MKN-3 (2), with a London dG value of −9.555, has a higher binding affinity than MKN-1 (1), with a London dG value of −9.134, for a CA molecule. The anti-HIV activity of MKN-3 (2) however, is remarkably lower than that of MKN-1 (1). The reason for this might be the poor cell membrane permeability of MKN-3 (2), which therefore could not penetrate the cells. In general, the hydrophobicity of compounds is correlated to their cell membrane permeability. The log *P* value of MKN-3 (2) is 0.27, which is markedly lower than that of MKN-1 (1) (log *P*: 2.97). We investigated the design of derivatives more hydrophobic than MKN-3 (2). For the design of MKN-3 (2) derivatives, the docking model of MKN-3 (2) and a CA protein was constructed, and a site in the MKN-3 (2) molecule, whose relationship with the interaction with a CA protein is unknown, was sought (Fig. 3). As a result, the amino group of the homopiperazine ring, which is marked by a red circle in Fig. 3, was found to fail to interact with a CA protein. Consequently, this amino group was modified by alkylation or amidation, and was converted into a methylene group. Six derivatives (17, 18, 19, TKB063 (25), 26 and 27), with octanoyl, 2-propylpentanoyl, 2-phenylacetyl, i-propyl, *n*-heptyl or benzyl groups, respectively on the secondary nitrogen atom of the homopiperazine ring of MKN-3 (2), were synthesized. A derivative (29), with a methylene group in place of the secondary amine of the homopiperazine ring, was prepared. All of these derivatives have log *P* values noticeably higher than that of MKN-3 (2), and showed significantly higher anti-HIV activity (EC_50_ < 15 μM) than MKN-3 (2) (EC_50_ = 42 μM). The single exception was 18, which has relatively high cytotoxicity (CC_50_ = 9.1 μM) and fails to show anti-HIV activity below 9.1 μM. It was assumed that the anti-HIV activity of these MKN-3 (2) derivatives was increased as a result of their improved hydrophobicity and cell membrane permeability. If the hydrophobicity of such derivatives is higher (log *P* > 2), the cytotoxicity is higher (CC_50_ < 10 μM). In compound 18, the 2-propylpentanoyl group on the secondary nitrogen atom of the homopiperazine ring led to an increase in its hydrophobicity (log *P* = 2.81) and also strong cytotoxicity. Compounds 17, 26 and 27, with octanoyl, *n*-heptyl or benzyl groups on the secondary nitrogen atom of the homopiperazine ring, respectively, also caused an increase in hydrophobicity and strong cytotoxicity (log *P* = 2.66, 3.14, and 2.38, and CC_50_ = 8.8 μM, 4.4 μM, and 9.0 μM) although the same compounds (17, 26 and 27) showed high anti-HIV activity with EC_50_ = 6.0, 2.6 and 2.4 μM, respectively. This suggests that derivatives with appropriate log *P* values (1 < log *P* < 2) have a suitable balance of anti-HIV activity and cytotoxicity. Compounds 19 and 29, with a 2-phenylacetyl group on the secondary nitrogen atom of the homopiperazine ring and a methylene group instead of the secondary amine, exhibited moderate anti-HIV activity (EC_50_ = 15 μM and 10 μM) and slight cytotoxicity (CC_50_ = 33 μM and 32 μM), although they have appropriate log *P* values (1.76 and 1.94), respectively. TKB063 (25), with EC_50_ = 4.5 μM, CC_50_ > 50 μM, is the most effective of these compounds, indicating that i-propyl is suitable as a modifying group (log *P* = 1.31) on the secondary nitrogen atom of the homopiperazine ring of MKN-3 (2). Compared to MKN-1 (1), the anti-HIV activity of TKB063 (25) is slightly lower, but the cytotoxicity is significantly lower. Thus, TKB063 (25) can be seen to have some advantages.

**Table 2:** log *P* values, anti-HIV activity and cytotoxicity of MKN-3 (2) and its derivatives

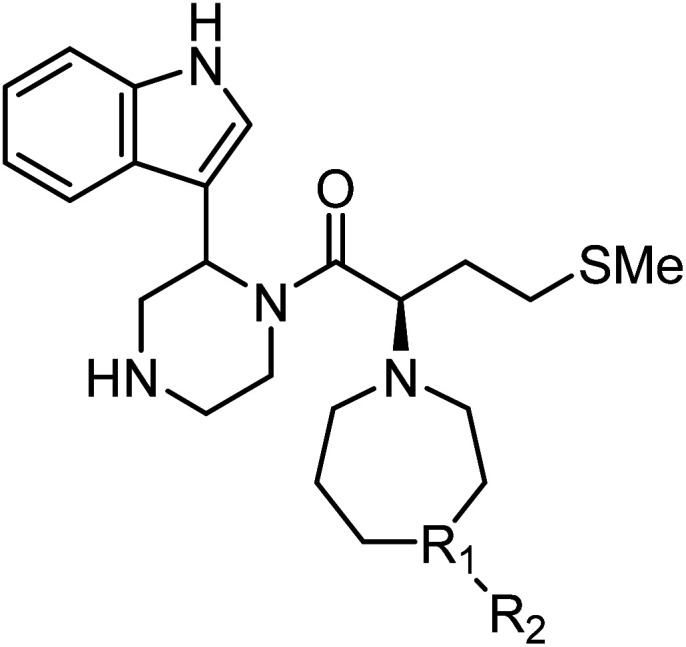					
Compound	R_1_	R_2_	log *P*^a^	EC_50_ (μM)^b^	CC_50_ (μM)^c^
MKN-3 (2)^d^	N	H	0.27	42	>50
17^d^	N	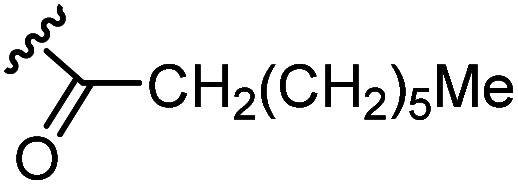	2.66	6.0	8.8
18^d^	N	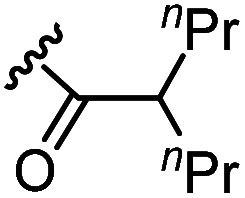	2.81	>9.1	9.1
19^d^	N	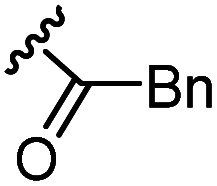	1.76	15	33
TKB063 (25)^d^	N	^i^Pr	1.31	4.5	>50
26^d^	N	*^n^* Heptyl	3.14	2.6	4.4
27^d^	N	Bn	2.38	2.4	9.0
29^d^	C	H_2_	1.94	10	32
MKN-1 (1)	—	—	2.97	2.2	15
AZT	—	—	—	0.021	67

a Calculated using ChemDraw Professional 15.1.

b EC_50_ values are the concentrations for 50% protection from HIV-1 (NL4-3 strain)-induced cytopathogenicity in MT-4 cells.

c CC_50_ values are the concentrations for 50% reduction of the viability of MT-4 cells. The data are a mean value of at least two independent experiments.

d diastereomixture.

TKB063 (25) is a mixture of its diastereomers. To investigate the anti-HIV activity of each of the diasteroisomers, they were separated by preparative RP-HPLC to yield TKB063A (25A) and TKB063B (25B) (Table 3). TKB063A (25A) and TKB063B (25B) showed almost the same anti-HIV activity, suggesting that both of the diastereoisomers have significant binding affinity to the CA protein and that the diastereomixture can be used for the evaluation of these derivatives. The assay data is consistent with the experimental results, docking simulations of the CA protein and the diastereoisomers of TKB063A (25A) and TKB063B (25B) using the Dock tool in MOE^80^ gave similar docking scores (TKB063A (25A): −5.92; TKB063B (25B): −5.85 kcal mol^−1^).

**Table 3:** Anti-HIV activity and cytotoxicity of the diastereoisomers of TKB063 (25)

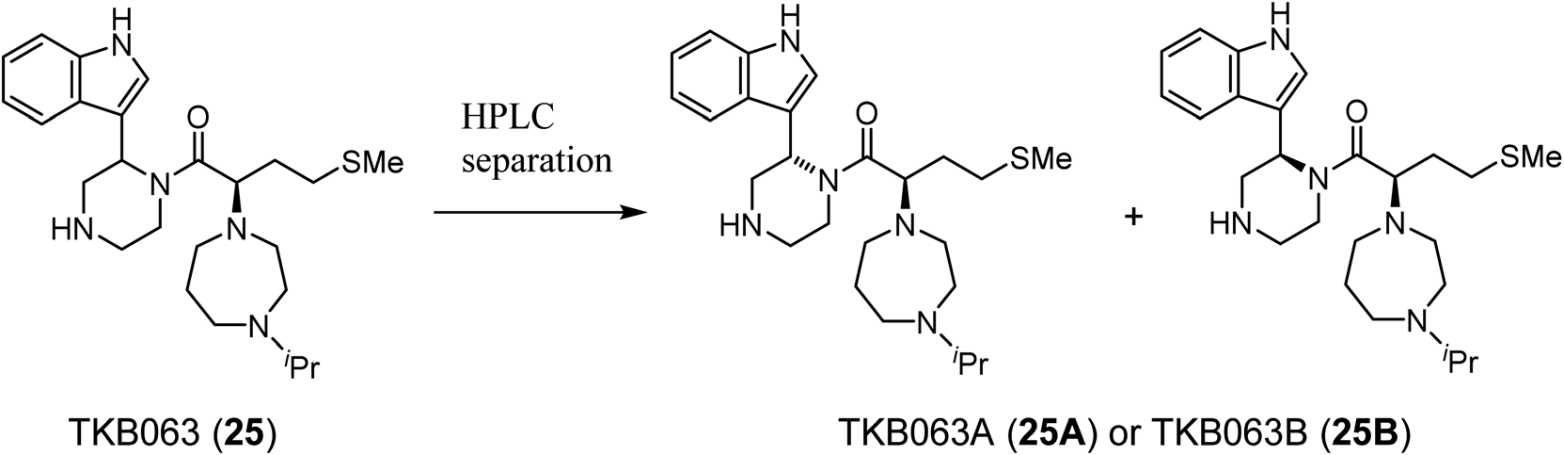		
Compound	EC_50_ (μM)^a^	CC_50_ (μM)^b^
TKB063 (25)^c^	4.8	>50
TKB063A (25A)	5.0	>50
TKB063B (25B)	7.4	>50
MKN-3 (2)^c^	>50	>50
MKN-1 (1)	2.3	15
AZT	0.025	>50

a EC_50_ values are the concentrations for 50% protection from HIV-1 (NL4-3 strain)-induced cytopathogenicity in MT-4 cells.

b CC_50_ values are the concentrations for 50% reduction of the viability of MT-4 cells. The data are a mean value of at least two independent experiments.

c Diastereomixture.

Next, additional derivatives with log *P* values (*ca.* 1 < log *P* < 2) were designed and synthesized as their diastereomixtures. Three derivatives, TON01 (36), TON02 (37) and TON03 (38), with ethyl, *n*-propyl or *sec*-butyl groups, respectively, on the secondary nitrogen atom of the homopiperazine ring of MKN-3 (2), were synthesized. These derivatives have log *P* values (0.99 < log *P* < 1.8), and show significant anti-HIV activity (EC_50_ < 20 μM) but no significant cytotoxicity (CC_50_ > 50 μM) (Table 4). This confirmed that derivatives with appropriate log *P* values (0.99 < log *P* < 1.8) have a suitable balance of anti-HIV activity and cytotoxicity due to appropriate hydrophobicity and cell membrane permeability shown in Table 2. TON02 (37) and TON03 (38) with EC_50_ = 8.0 μM, 6.3 μM, respectively, are the most effective derivatives among these compounds. Both derivatives are comparable with TKB063 (25). This suggests that *n*-propyl and *sec*-butyl are suitable as modifying groups (log *P* = 1.48 and 1.79) similar to an i-propyl group in TKB063 (25) on the secondary nitrogen atom of the homopiperazine ring of MKN-3 (2). Cytotoxicity of TON02 (37) and TON03 (38) was not observed below 50 μM (CC_50_ > 50 μM) similar to that of TKB063 (25). An ethyl group might be responsible for the small size and low hydrophobicity (log *P* = 0.99) because TON01 (36) showed lower antiviral activity (EC_50_ = 20 μM) than TON02 (37), TON03 (38) or TKB063 (25).

**Table 4:** log *P* values, anti-HIV activity and cytotoxicity of additional derivatives

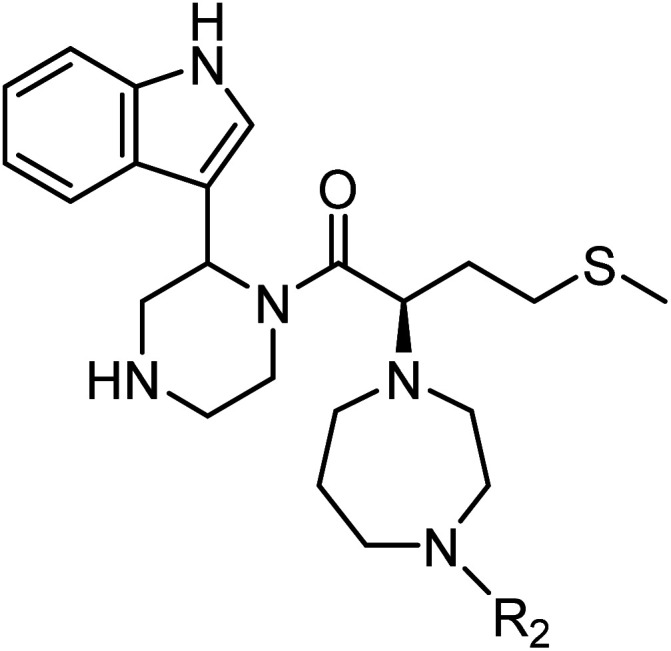				
Compound	R_2_	log *P*^a^	EC_50_ (μM)^b^	CC_50_ (μM)^c^
TON01 (36)^d^	Et	0.99	20	>50
TON02 (37)^d^	^*n*^Pr	1.48	8.0	>50
TON03 (38)^d^	^*s*^Bu	1.79	6.3	>50
TKB063 (25)^d^	^i^Pr	1.31	9.5	>50
AZT	—	—	0.040	>50

a Calculated using ChemDraw Professional 15.1.

b EC_50_ values are the concentrations for 50% protection from HIV-1 (NL4-3 strain)-induced cytopathogenicity in MT-4 cells.

c CC_50_ values are the concentrations for 50% reduction of the viability of MT-4 cells. The data are a mean value of at least two independent experiments.

d Diastereomixture.

## Conclusion

In this study, MKN-3 (2) derivatives, a new class of small molecules, with a scaffold structure different from that of MKN-1 (1), were developed based on *in silico* screening as a dipeptide mimic of Trp184 and Met185 at the hydrophobic interaction site between two CA molecules that are important for stabilization of the multimeric structure of CA. The original compound MKN-3 (2), failed however to show higher anti-HIV activity than MKN-1 (1), possibly due to its low hydrophobicity and lack of cell membrane permeability of MKN-3 (2). Derivatives more hydrophobic than MKN-3 (2) were designed using the docking model of MKN-3 (2) with a CA protein (Fig. 3). The amino group of the homopiperazine ring does not interact with a CA protein, and accordingly, this amino group was modified. Three derivatives, TKB063 (25), TON02 (37) and TON03 (38), with an i-propyl, *n*-propyl or *sec*-butyl group on the secondary nitrogen atom of the homopiperazine ring of MKN-3 (2), respectively, were prepared and showed high anti-HIV activity. The two diastereomers of TKB063 (25), TKB063A (25A) and TKB063B (25B), showed almost the same anti-HIV activity, suggesting that both have significant binding affinity for the CA protein, and that the diastereomixture can be used in the evaluation of these derivatives. Compared to our previous lead compound, MKN-1 (1), the anti-HIV activity of these MKN-3 (2) derivatives is slightly lower, but the cytotoxicity is significantly lower. These are some advantages. The present data will be useful and important in the future design of new anti-HIV agents with action mechanisms different from existing.

## Experimental

### *In silico* screening of antiviral candidates^75^

In this study, we attempted to design compounds that could impact on CA dimerization in addition to the interactions among CA hexamers, and CA–CA interactions in the maturation of core. To perform the *in silico* screening, we first obtained the structure of the dimer of CA proteins (PDB ID: 3J34) from the Protein Data Bank (https://www.rcsb.org/). The structure of the dimer of CA proteins was thermodynamically optimized by energy minimization using MOE^80^ and the Amber10:EHT force field.^81,82^ One monomer of the dimer of CA proteins was fixed as a receptor, and the residues of the other monomer with the exception of the Typ184 and Met185 residues, were removed, their side chains playing a key role in the dimer formation. Using a complex composed of the CA monomer as a receptor and Trp184–Met185 dipeptide unit as a ligand, we searched for the compounds having a higher affinity for the monomer than was observed in the dimer formation. To do this, replacement of the main chain backbone of the dipeptide on the CA protein was performed by the Scaffold Replacement application in MOE using the linker database of the MOE and the Amber10:EHT force field, while the side chains of the dipeptide remained fixed. From the result, we selected the compounds with higher scores of the binding affinity for receptor (London dG), ligand efficacy, and topological polar surface area (TPSA). Docking simulation of the CA protein and the diastereoisomers of TKB063A (25A) and TKB063B (25B) was done using the Dock tool in MOE.^80^ Briefly, the CA monomer model used for designing TKB063 was fixed as a receptor for ligand binding, while TKB063A (25A) or TKB063B (25B) was defined as a ligand for the CA binding. The docking simulations were executed to collect physicochemically possible docking poses between the receptor and ligand moieties using the Dock tool in MOE. Minimal docking scores of TKB063A (25A) or TKB063B (25B) were used for the comparison of binding affinity.

### Synthesis of MKN-3 (2) and its derivatives

The synthetic methods for MKN-3 (2) and its derivatives are described in Schemes 2–6. The purity of all of the final compounds, measured by analytical HPLC or NMR is >95%. Experimental procedures including characterization data are provided in the ESI.†

### Evaluation of anti-HIV-1 activity and cytotoxicity^75^

For virus preparation, 293T/17 cells (Invitrogen) maintained in Dulbecco's modified Eagle medium (DMEM) containing 10% FBS, in a T-75 flask were transfected with 10 μg of the pNL4-3 construct by the calcium phosphate method. The supernatant was collected 48 h after transfection, passed through a 0.45 μm filter, and stored at −80 °C as a stock virus.

The inhibitory activity of test compounds against X4-HIV-1 (NL4-3 strain)-induced cytopathogenicity in MT-4 cells,^83^ which are maintained in RPMI-1640 containing 10% FBS, was assessed by an MTT assay. Various concentrations of test compound solutions were added to HIV-1-infected MT-4 cells at multiplicity of infection (MOI) of 0.001, and placed in wells of a 96-well microplate. The test compounds and the reference compounds, *e.g.* AZT, were diluted by factors of two or five, respectively. After 5 days' incubation at 37 °C in a CO_2_ incubator, the number of viable cells was determined by the MTT assay. Cytotoxicities of the test compounds were determined based on reduction of the viability of MT-4 cells measured in the MTT assay. A reverse transcriptase inhibitor, AZT, was purchased from Sigma Aldrich and employed as a positive control compound with anti-HIV activity.

## Author contributions

T. K., M. Y., K. T., M. F., M. K., T. O., S. B., T. I., Y. M., N. O., O. K.: investigation; M. Y., O. K.: analysis; T. M., H. S., H. T.: supervision; T. K., M. Y., K. T., K. S., Y. K., T. M., H. S., H. T.: writing. All authors have given approval to the final version of the manuscript.

## Conflicts of interest

The authors declare no conflict of interest.

## Supplementary Material

RA-013-D2RA06837K-s001
