# Supplementary material for: Low-molecular-weight anti-HIV-1 agents targeting HIV-1 capsid proteins
Source: RSC Adv. 2023 Jan 12;13(3):2156–67. doi: 10.1039/d2ra06837k (PMC9834766; doi:10.1039/d2ra06837k)
Supplement: RA-013-D2RA06837K-s001 [file RA-013-D2RA06837K-s001.pdf]

Electronic Supplementary Material (ESI) for RSC Advances.
This journal is © The Royal Society of Chemistry 2023

Electronic supplementary information (ESI)

Low-molecular-weight Anti-HIV-1 Agents Targeting HIV-1 Capsid Proteins

Takuya Kobayakawa,[a] Masaru Yokoyama,[b] Kohei Tsuji,[a] Masayuki Fujino,[c] Masaki Kurakami,[a] Takato Onishi,[a] Sayaka Boku,[a] Takahiro Ishii,[a] Yutaro Miura,[a] Kouki Shinohara,[a] Yuki Kishihara,[a] Nami Ohashi,[d] Osamu Kotani,[b] Tsutomu Murakami,*[c] Hironori Sato*[b] and Hirokazu Tamamura*[a]

[a]*Institute of Biomaterials and Bioengineering, Tokyo Medical and Dental University (TMDU), 2-3-10 Kandasurugadai, Chiyoda-ku, Tokyo 101-0062, Japan. E-mail: tamamura.mr@tmd.ac.jp*

[b]*Pathogen Genomics Center, National Institute of Infectious Diseases, Musashimurayama, Tokyo 208-0011, Japan. E-mail: hirosato@nih.go.jp*

[c]*AIDS Research Center, National Institute of Infectious Diseases, Shinjuku-ku, Tokyo 162-8640, Japan. E-mail: tmura@nih.go.jp*

[d]*Showa Pharmaceutical University, Machida, Tokyo 194-8543, Japan*

Table of contents

I. General information S2

I-I. General methods S2

I-II. Characterization Data S2

II. Experimental procedures S3

III. References S20

IV. ^{1}H NMR and ^{13}C NMR charts (compounds 25A, 25B and 38) S21

V. High Resolution Mass Spectrometry (HRMS) charts (compounds 25A, 25B and 38) S24

VI. HPLC charts (compounds 2, 17, 18, 19, 25A, 25B, 26, 27, 29, 36, 37 and 38) S27

I. General information

I-I. General methods

All reactions were performed using commercially supplied reagents and solvents in dried glassware under an atmosphere of nitrogen unless otherwise noted. Thin-layer chromatography (TLC) was performed on Merck $60F_{254}$ precoated silica gel plates and was visualized by fluorescence quenching under UV light and by staining with phosphomolybdic acid, *p*-anisaldehyde, or ninhydrin, respectively. Flash column chromatography was carried out with silica gel 60 N (Kanto Chemical Co., Inc.) or automatic silica gel flash column chromatography system (Isolera One (Biotage, Sweden). Preparative RP-HPLC was performed using a Cosmosil $5C_{18}$-ARII column (20 × 250 mm, Nacalai Tesque, Inc., Japan) on a JASCO PU-2089 plus (JASCO Corporation, Ltd., Japan) in a linear gradient of CH_3CN containing 0.1% TFA (Solvent B) in H_2O containing 0.1% (v/v) TFA (Solvent A) at a flow rate of 10 cm^3 min^{-1}, and eluting products were detected by UV at 254 nm.

I-II. Characterization Data

^1H NMR (400 MHz or 500 MHz) and ^{13}C NMR (100 MHz or 125 MHz) spectra were recorded using a Bruker AVANCE III 400 spectrometer and Bruker AVANCE 500 spectrometer (Bruker, USA). Coupling constants are reported in Hertz, and peak shifts are reported in d (ppm) relative to $CDCl_3$ (^1H 7.26 ppm, ^{13}C 77.16 ppm), MeOD (^1H 3.31 ppm, ^{13}C 49.00 ppm) or dimethyl sulfoxide (DMSO)-d_6 (^1H 2.50 ppm, ^{13}C 39.52 ppm). Low- and high-resolution mass spectra were recorded on a Bruker Daltonics micrOTOF focus in the positive and negative detection mode. For analytical RP-HPLC, a Cosmosil $5C_{18}$-ARII column (4.6 × 250 mm, Nacalai Tesque, Inc.) was employed with a linear gradient of CH_3CN containing 0.1% (v/v) trifluoroacetic acid (TFA) (Solvent B) in H_2O containing 0.1% (v/v) TFA (Solvent A) at a flow rate of 1.0 cm^3 min^{-1} on a PU-2089 plus (JASCO Corporation, Ltd.), and eluting products were detected by UV at 254 nm. Elemental analyses were performed by A Rabbit Science Japan Co., Ltd., Sagamihara, Kanagawa.

II. Experimental procedures

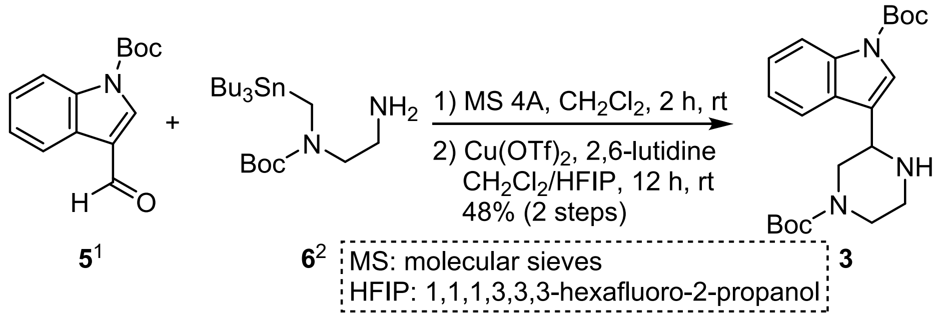

***tert*-Butyl 3-(4-(*tert*-butoxycarbonyl)piperazin-2-yl)-1*H*-indole-1-carboxylate** (**3**): Compound **6** and compound **3** were synthesized using previously reported procedure.[1,2] **5** (1.23 g, 5.00 mmol) and SnAP Pip **6** (209 µL, 5.00 mmol) was converted into the title compound **3** (1.91 g, 4.77 µmol, 48% in 2 steps) as a white solid; ^{1}H NMR (500 MHz, $CDCl_3$) δ 1.49 (s, 9H), 1.66 (s, 9H), 2.92-3.12 (m, 4H), 4.03-4.33 (m, 3H), 7.23-7.34 (m, 2H), 7.60 (s, 1H), 7.69 (s, 1H), 8.16 (s, 1H); ^{13}C{^{1}H} NMR (125 MHz, $CDCl_3$) δ 28.4 (3C), 28.6 (3C), 46.2 (2C), 52.8 (2C), 80.0, 83.8, 115.6, 119.4, 121.1, 122.7, 124.7, 129.0, 135.8, 149.8, 154.9; HRMS (ESI), *m*/*z* calcd for $C_{22}H_{32}N_3O_4$ $[M+H]^+$ 402.2387, found 402.2382.

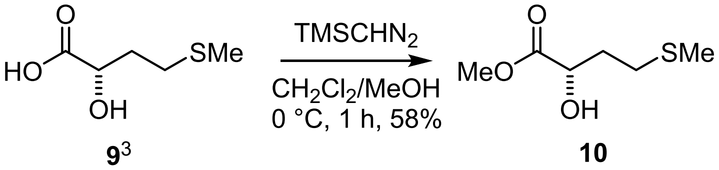

Methyl (*S*)-2-hydroxy-4-(methylthio)butanoate (**10**): Compound **9** was synthesized using previously reported procedure.[3] To a solution of **9** (5.57 g, 37.1 mmol) in MeOH (90.0 mL) and CH_2Cl_2 (90.0 mL) was added a solution of Trimethylsilyl (TMS) diazomethane in Et_2O (2.00 M, 28.0 mL, 55.7 mmol) at 0 °C. The reaction mixture was warmed to room temperature and stirred for 1 h. The reaction was quenched by the addition of saturated aqueous NH_4Cl and extracted with EtOAc. The organic layer was dried over $MgSO_4$ and concentrated under reduced pressure followed by purification using flash column chromatography with *n*-hexane/EtOAc (3:1) to obtain the title compound **10** (3.53 g, 58% yield) as yellow oil; ^{1}H NMR (400 MHz, $CDCl_3$) δ 1.88-2.14 (m, 2H), 2.11 (s, 3H), 2.62-2.66 (m, 2H), 3.08 (s, 3H), 4.32-4.35 (m, 1H); ^{13}C{^{1}H} NMR (125 MHz, $CDCl_3$) δ 15.6, 29.7, 33.6, 52.8, 69.3, 175.5; HRMS (ESI), *m*/*z* calcd for $C_6H_{12}NaO_3S$ $[M+Na]^+$ 187.0399, found 187.0404.

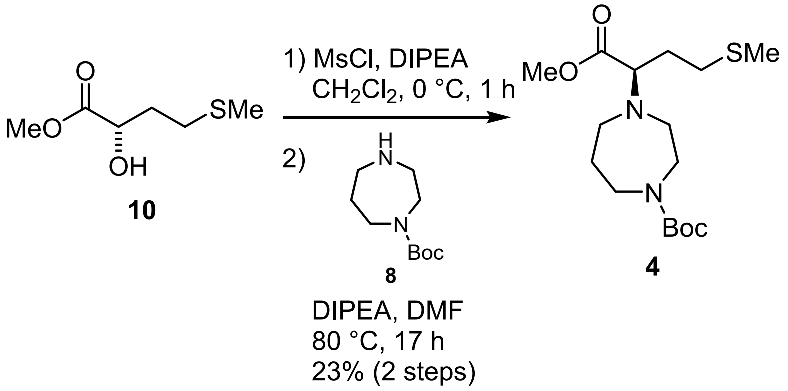

***tert*-Butyl (*R*)-4-(1-methoxy-4-(methylthio)-1-oxobutan-2-yl)-1,4-diazepane-1-carboxylate** (**4**): To a solution of **10** (4.96 g, 30.2 mmol) and *N*,*N*-diisopropylethylamine (DIPEA, 15.8 mL, 90.6 mmol) in CH_2Cl_2 (150 mL) was added mesyl chloride (MsCl, 4.00 mL, 45.3 mmol) at 0 °C. The reaction mixture was warmed to room temperature and stirred for 1 h. The reaction mixture was added saturated aqueous NH_4Cl. The mixture was extracted with CH_2Cl_2 and washed with brine. The organic layer was dried over $MgSO_4$ and concentrated under reduced pressure to provide the crude mesylate. The crude mesylate and DIPEA (15.8 mL, 90.6 mmol) in *N*,*N*-dimethylformamide (DMF, 60.0 mL) were added *tert*-butyl 1,4-diazepane-1-carboxylate **8** (12.1 g, 60.4 mmol) at room temperature. The reaction mixture was stirred at 80 °C for 17 h. The reaction was quenched by the addition of saturated aqueous NH_4Cl and the mixture was extracted with EtOAc. The organic layer was dried over $MgSO_4$ and concentrated under reduced pressure followed by purification using flash column chromatography with *n*-hexane/EtOAc (3:1) to obtain the title compound **4** (2.41 g, 23% in 2 steps) as yellow oil: ^{1}H NMR (500 MHz, $CDCl_3$): (mixture of rotamers) δ 1.45 (s, 9H), 1.69-1.82, (m, 1H), 1.84-2.01 (m, 2H), 2.09 (s, 3H), 2.56-2.72 (m, 4H), 2.80-2.91 (m, 2H), 3.33-3.50 (m, 5H), 3.69 (m, 3H); $^{13}C\{^1H\}$ NMR (125 MHz, $CDCl_3$): (mixture of rotamers) δ 15.4, 28.5 (3C), 29.1, 29.3, 30.9, 46.1, 48.4, 51.2, 51.8, 52.9, 66.5, 66.6, 79.2, 155.5, 173.3; HRMS (ESI), *m*/*z* calcd for $C_{16}H_{30}N_2O_4S$ $[M+H]^+$ 347.1999, found 347.2001.

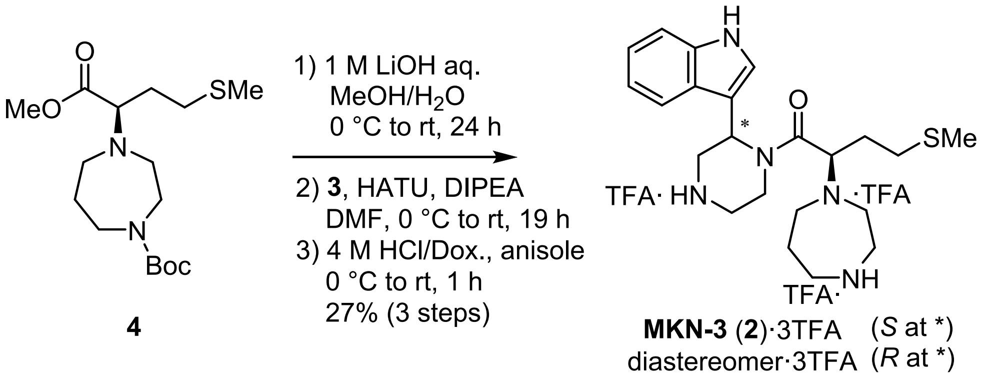

(2*R*)-1-(2-(1*H*-Indol-3-yl)piperazin-1-yl)-2-(1,4-diazepan-1-yl)-4-(methylthio)butan-1-one·3TFA (**2**·3TFA, **MKN-3**·3TFA): To a solution of **4** (34.6 mg, 100 µmol) in MeOH (1.00 mL) was added 1.00 M LiOH aq. (250 µL, 250 µmol) at 0 °C. The reaction mixture was warmed to room temperature and stirred for 24 h. The reaction mixture was added saturated aqueous NH_4Cl and extracted with $CHCl_3$. The organic layer was dried

over $MgSO_4$ and concentrated under reduced pressure to provide the crude carboxylic acid. The carboxylic acid in DMF (1.00 mL) was added 1-[bis(dimethylamino)methylene]-1*H*-1,2,3-triazolo[4,5-*b*]pyridinium 3-oxide hexafluorophosphate (HATU, 41.8 mg, 110 μmol), DIPEA (105 μL, 600 μmol) and **3** (44.2 mg, 110 μmol) at 0 °C. The reaction mixture was warmed to room temperature and stirred for 19 h. The reaction was quenched by the addition of saturated aqueous NH_4Cl and the mixture was extracted with EtOAc. The organic layer was dried over $MgSO_4$ and concentrated under reduced pressure to obtain the crude amide. The crude amide and anisole (130 μL, 1.20 mmol) in CH_2Cl_2 (500 μL) was added 4 M HCl/dioxane (HCl/Dox., 500 μL, 2.00 mmol) at 0 °C. The reaction mixture was stirred for 1 h at room temperature. The mixture was concentrated under reduced pressure followed by purification with preparative RP-HPLC to obtain the *tris*-trifluoroacetate salt of the title compound **2** (20.6 mg, 27.2 μmol, 27% in 3 steps) as freeze-dried powder: t_R = 21.6 min and 22.1 min (linear gradient of B in A, 10 to 30% over 40 min); ^{1}H NMR (500 MHz, DMSO-d_6): (mixture of diastereomers) δ 1.76-2.09 (m, 6H), 2.73-3.39 (m, 10H), 3.90-4.24 (m, 7H), 5.75-6.18 (m, 1H), 6.91-7.06 (m, 1H, indole C5,C6-H), 7.08-7.12 (m, 1H, indole C5,C6-H), 7.32-7.52 (m, 2H, indole C2-H and C4,C7-H), 7.53-7.64 (m, 1H, indole C4,C7-H), 8.61-8.79 (m, 1H), 9.49-9.62 (m, 1H), 11.3 (br, 1H, indole N-H); ^{13}C{^{1}H} NMR (125 MHz, DMSO-d_6): (mixture of diastereomers) δ 15.1, 24.9, 25.0, 25.4, 25.9, 30.8, 30.9, 31.0, 38.6, 44.4, 45.3, 47.2, 50.8, 63.2, 109.6, 112.2, 115.9, 118.3, 119.1, 122.0, 125.1, 137.1, 158.7; HRMS (ESI), *m/z* calcd for $C_{22}H_{33}N_5OS$ $[M+H]^+$ 416.2479, found 416.2479. Anal. Calcd for $C_{22}H_{32}N_5OS\cdot3TFA$: C, 44.39; H, 4.79; N, 9.24. Found: C, 45.23; H, 5.67; N, 9.47.

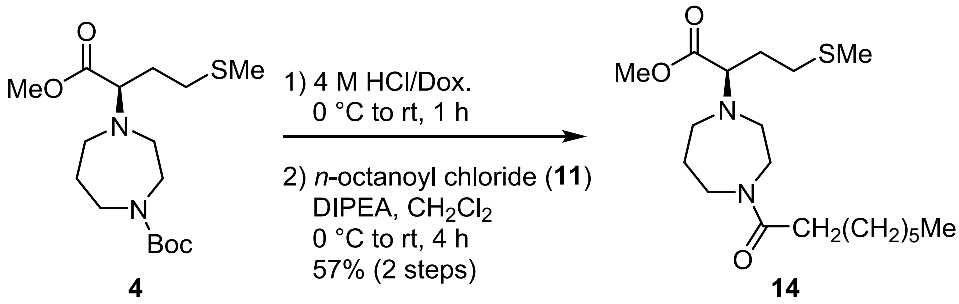

Methyl (*R*)-4-(methylthio)-2-(4-octanoyl-1,4-diazepan-1-yl)butanoate (**14**): Compound **4** (173 mg, 500 μmol) was added 4 M HCl/dioxane (2.50 mL, 10.0 mmol) at 0 °C. The reaction mixture was stirred at room temperature for 1 h. The mixture was concentrated under reduced pressure to obtain the crude amine. The crude amine in CH_2Cl_2 (2.50 mL) was added DIPEA (348 μL, 2.00 mmol) and *n*-octanoyl chloride **11** (173 μg, 1.00 mmol) at room temperature. The reaction mixture was warmed to room temperature and stirred for 4 h. The reaction mixture was added silica gel and evaporated. The residue was purified by automated silica gel flush column chromatography system (Isolera One) with *n*-hexane/EtOAc (92:8 to 30:70) to obtain the title compound **14** (102 mg, 57% in 2 steps) as colorless oil: ^{1}H NMR (500 MHz, $CDCl_3$): (mixture of rotamers) δ 0.86-0.89 (m, 3H), 1.28-1.31 (m, 8H), 1.62-1.63 (m, 2H), 1.69-2.03 (m, 4H), 2.09 (s, 3H), 2.27-2.31 (m, 2H), 2.56-2.98 (m, 6H), 3.44-3.62 (m, 5H), 3.68-3.70 (m, 3H); ^{13}C{^{1}H} NMR (125 MHz, $CDCl_3$): (mixture of rotamers) δ 14.2, 15.6, 22.8, 25.5, 29.3, 29.7, 31.1, 31.9, 33.6, 44.6, 47.2, 49.7, 51.5, 52.0, 52.7, 54.0, 66.7, 173.0, 173.3; HRMS

(ESI), *m/z* calcd for $C_{19}H_{37}N_2O_3S$ $[M+H]^+$ 373.2519, found 373.2514.

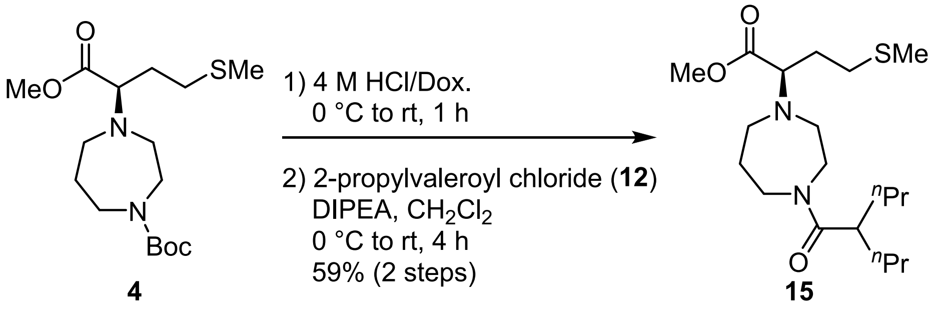

Methyl (*R*)-4-(methylthio)-2-(4-(2-propylpentanoyl)-1,4-diazepan-1-yl)butanoate (**15**): Compound **4** (173 mg, 500 μmol) was added 4 M HCl/dioxane (2.50 mL, 10.0 mmol) at 0 °C. The reaction mixture was stirred at room temperature for 1 h, and then concentrated under reduced pressure to obtain the crude amine. The crude amine in CH_2Cl_2 (2.50 mL) was added DIPEA (348 μL, 2.00 mmol) and 2-propylvaleroyl chloride **12** (171 μL, 1.00 mmol) at room temperature. The reaction mixture was warmed to room temperature and stirred for 4 h. The reaction mixture was added silica gel and evaporated. The residue was purified by automated silica gel flush column chromatography system (Isolera One) with *n*-hexane/EtOAc (92:8 to 30:70) to obtain the title compound **15** (106 mg, 59% in 2 steps) as colorless oil: ^1H NMR (500 MHz, $CDCl_3$): (mixture of rotamers) δ 0.89 (t, *J* = 7.5 Hz, 6H), 1.21-1.41 (m, 6H), 1.62-2.04 (m, 6H), 2.09 (s, 3H), 2.56-2.98 (m, 7H), 3.49-3.64 (m, 4H), 3.68-3.69 (m, 3H); $^{13}C\{^1H\}$ NMR (125 MHz, $CDCl_3$): (mixture of rotamers) δ 14.5, 15.5, 21.1, 28.7, 29.4, 30.2, 31.1, 35.5, 35.6, 41.2, 44.6, 47.0, 49.5, 51.4, 53.3, 54.4, 66.7, 173.2, 176.1; HRMS (ESI), *m/z* calcd for $C_{19}H_{36}N_2O_3S$ $[M+H]^+$ 373.2519, found 373.2514.

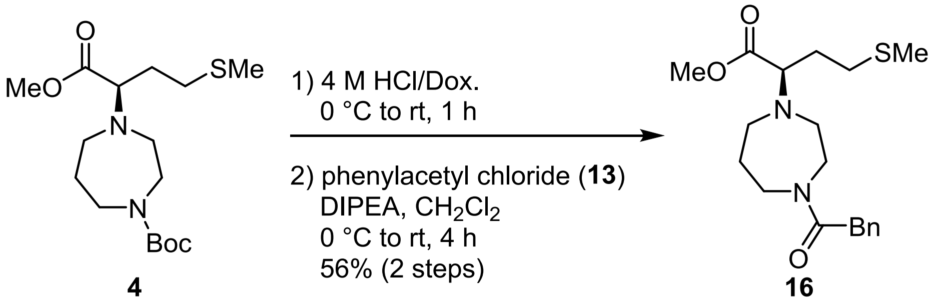

Methyl (*R*)-4-(methylthio)-2-(4-(2-phenylacetyl)-1,4-diazepan-1-yl)butanoate (**16**): Compound **4** (173 mg, 500 μmol) was added 4 M HCl/dioxane (2.50 mL, 10.0 mmol) at 0 °C. The reaction mixture was stirred at room temperature for 1 h, and then concentrated under reduced pressure to obtain the crude amine. The crude amine in CH_2Cl_2 (2.50 mL) was added DIPEA (348 μL, 2.00 mmol) and phenylacetyl chloride **13** (132 μL, 1.00 mmol) at room temperature. The reaction mixture was stirred at room temperature for 4 h. The reaction mixture was added silica gel and evaporated. The residue was purified by automated silica gel flush column chromatography system (Isolera One) with *n*-hexane/EtOAc (88:12 to 0:100) to obtain the title compound **16** (103 mg, 56% in

2 steps) as brown oil: ^{1}H NMR (500 MHz, $CDCl_3$): (mixture of rotamers) δ 1.66-1.76 (m, 1H), 1.77-1.91 (m, 2H), 1.92-2.01 (m, 1H), 2.08-2.09 (m, 3H), 2.50-2.91 (m, 6H), 3.44-3.72 (m, 10H), 7.22-7.33 (m, 5H); $^{13}C\{^{1}H\}$ NMR (125 MHz, $CDCl_3$): (mixture of rotamers) δ 15.6, 28.3, 29.3, 31.0, 41.4, 44.8, 47.4, 50.1, 51.9, 51.5, 52.0, 53.8, 66.7, 126.9, 128.8, 128.9, 135.3, 170.8, 173.2; HRMS (ESI), *m/z* calcd for $C_{19}H_{29}N_2O_3S$ $[M+H]^+$ 365.1893, found 365.1888.

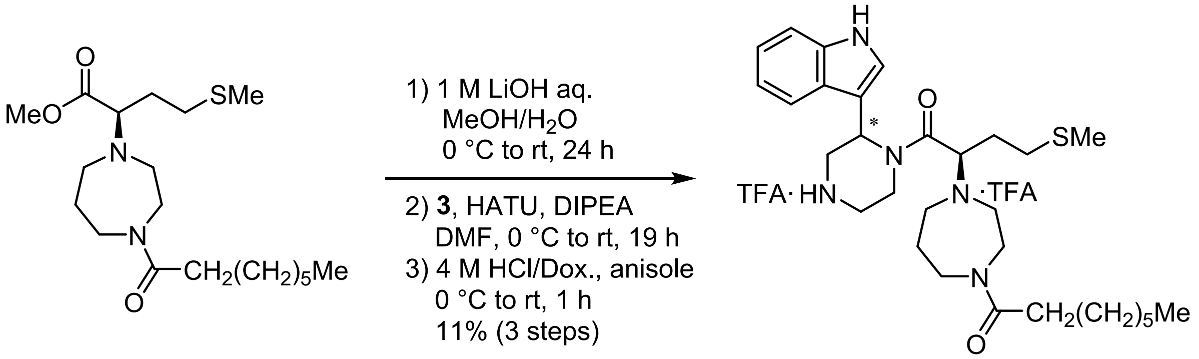

14 **17**·2TFA

1-(4-((2*R*)-1-(2-(1*H*-Indol-3-yl)piperazin-1-yl)-4-(methylthio)-1-oxobutan-2-yl)-1,4-diazepan-1-yl)-2-propylpentan-1-one·2TFA (**17**·2TFA): To a solution of compound **14** (35.9 mg, 100 μmol) in MeOH (1.00 mL) was added 1.00 M LiOH aq. (250 μL, 250 μmol) at 0 °C. The reaction mixture was stirred at room temperature for 24 h. The reaction was quenched by the addition of saturated aqueous NH_4Cl and extracted with $CHCl_3$. The organic layer was dried over $MgSO_4$ and concentrated under reduced pressure to obtain the crude carboxylic acid. The crude carboxylic acid in DMF (1.00 mL) was added HATU (41.8 mg, 110 μmol), DIPEA (105 μL, 600 μmol) and compound **3** (44.2 mg, 110 μmol) at 0 °C. The reaction mixture was stirred at room temperature for 19 h. The mixture was added saturated aqueous NH_4Cl and extracted with EtOAc. The organic layer was dried over $MgSO_4$ and concentrated under reduced pressure to obtain the crude amide. The crude amide and anisole (109 μL, 1.00 mmol) in CH_2Cl_2 (500 μL) were added 4 M HCl/dioxane (500 μL, 2.00 mmol) at 0 °C. The reaction mixture was stirred for 1 h at room temperature, and then concentrated under reduced pressure followed by purification with preparative RP-HPLC to obtain the *bis*-trifluoroacetate salt of the title compound **17** (8.30 mg, 10.8 μmol, 11% in 3 steps) as freeze-dried powder: t_R = 22.5 min and 22.8 min (linear gradient of B in A, 20 to 50% over 30 min); ^{1}H NMR (500 MHz, MeOD): (mixture of diastereomers and rotamers) δ 0.89-0.92 (m, 3H), 1.17-3.22 (m, 28H), 3.41-4.38 (m, 9H), 6.96-7.11 (m, 1H, indole C5,C6-H), 7.13-7.23 (m, 1H, indole C5,C6-H), 7.29-7.65 (m, 3H, indole C2-H and C4,C7-H); $^{13}C\{^{1}H\}$ NMR (125 MHz, MeOD): (mixture of diastereomers and rotamers) δ 15.1, 15.2, 15.3, 23.7, 24.6, 26.6, 29.3, 30.3, 30.5, 32.1, 32.3, 32.9, 34.2, 39.6, 44.5, 45.2, 45.9, 46.7, 47.5, 65.7, 112.9, 119.7, 120.7, 123.4, 123.5, 124.8, 124.9, 138.7, 162.9, 175.7; HRMS (ESI), *m/z* calcd for $C_{30}H_{48}N_5O_2S$ $[M+H]^+$ 542.3523, found 542.3523. Anal. Calcd for $C_{30}H_{47}N_5O_2S$·2TFA: C, 53.05; H, 6.42; N, 9.10. Found: C, 53.14; H, 6.71; N, 8.74.

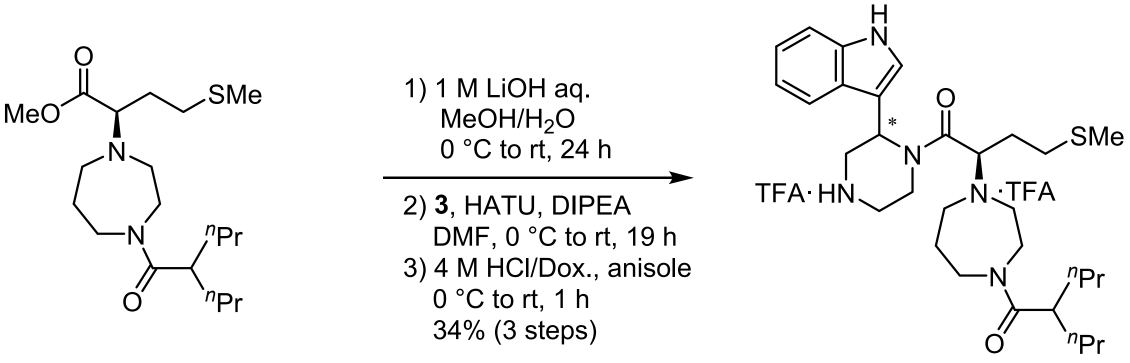

15 **18**·2TFA

1-(4-((2*R*)-1-(2-(1*H*-Indol-3-yl)piperazin-1-yl)-4-(methylthio)-1-oxobutan-2-yl)-1,4-diazepan-1-yl)octan-1-one·2TFA (**18**·2TFA): To a solution of compound **15** (35.9 mg, 100 μmol) in MeOH (1.00 mL) was added 1.00 M LiOH aq. (250 μL, 250 μmol) at 0 °C. The reaction mixture was stirred at room temperature for 24 h. The reaction was quenched by the addition of saturated aqueous NH_4Cl and the mixture was extracted with $CHCl_3$. The organic layer was dried over $MgSO_4$ and concentrated under reduced pressure to obtain the crude carboxylic acid. The crude carboxylic acid in DMF (1.00 mL) was added HATU (41.8 mg, 110 μmol), DIPEA (105 μL, 600 μmol) and compound **3** (44.2 mg, 110 μmol) at 0 °C. The reaction mixture was stirred at room temperature for 19 h. The reaction was quenched by the addition of saturated aqueous NH_4Cl and the mixture was extracted with EtOAc. The organic layer was dried over $MgSO_4$ and concentrated under reduced pressure to obtain the crude amide. The crude amide and anisole (109 μL, 1.00 mmol) in CH_2Cl_2 (500 μL) were added 4 M HCl/dioxane (500 μL, 2.00 mmol) at 0 °C. The reaction mixture was stirred for 1 h at room temperature, and then concentrated under reduced pressure followed by purification with preparative RP-HPLC to obtain the *bis*-trifluoroacetate salt of the title compound **18** (26.4 mg, 34.0 μmol, 34% in 3 steps) as freeze-dried powder: t_R = 20.0 min and 20.7 min (linear gradient of B in A, 20 to 50% over 30 min); ^{1}H NMR (500 MHz, MeOD): (mixture of diastereomers and rotamers) δ 0.72-1.02 (m, 6H), 1.09-3.29 (m, 25H), 3.35-4.53 (m, 9H), 6.99-7.13 (m, 1H, indole C5,C6-H), 7.14-7.26 (m, 1H, indole C5,C6-H), 7.32-7.66 (m, 3H, indole C2-H and C4,C7-H), 10.93 (br, 1H, indole N-H); ^{13}C{^{1}H} NMR (125 MHz, MeOD): (mixture of diastereomers and rotamers) δ 14.6, 14.7, 21.8, 21.9, 27.7, 31.3, 36.4, 36.5, 39.7, 42.2, 44.1, 44.3, 45.0, 45.3, 45.9, 46.5, 46.9, 49.9, 54.2, 65.6, 112.9, 113.1, 119.7, 120.7, 123.5, 125.0, 126.7, 138.6, 162.8, 178.5; HRMS (ESI), *m/z* calcd for $C_{30}H_{48}N_5O_2S$ $[M+H]^+$ 542.3523, found 542.3521. Anal. Calcd for $C_{30}H_{47}N_5O_2S$·2TFA: C, 53.05; H, 6.42; N, 9.10. Found: C, 50.38; H, 6.44; N, 8.67.

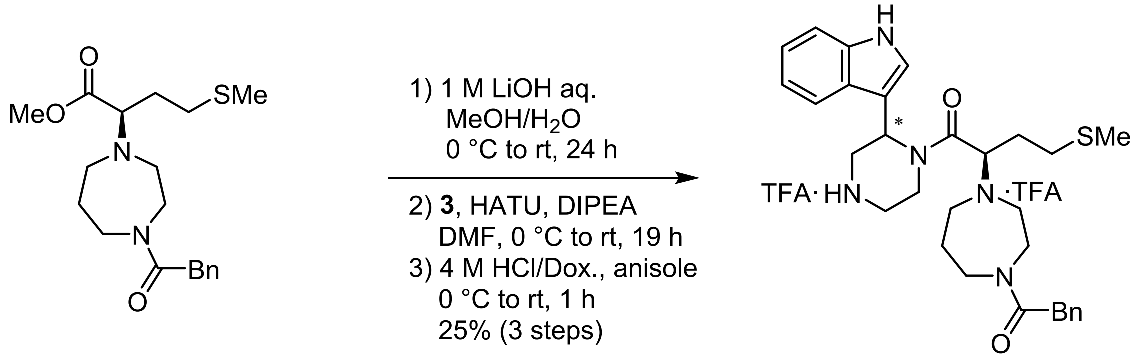

16 **19**·2TFA

(2*R*)-1-(2-(1*H*-Indol-3-yl)piperazin-1-yl)-4-(methylthio)-2-(4-(2-phenylacetyl)-1,4-diazepan-1-yl)butan-1-one·2TFA (**19**·2TFA): To a solution of compound **16** (36.5 mg, 0.100 mmol) in MeOH (1.00 mL) was added 1.00 M LiOH aq. (250 µL, 250 µmol) at 0 °C. The reaction mixture was stirred at room temperature for 24 h. The reaction was quenched by the addition of saturated aqueous NH_4Cl and the mixture was extracted with $CHCl_3$. The organic layer was dried over $MgSO_4$ and concentrated under reduced pressure to obtain the crude carboxylic acid. The crude carboxylic acid in DMF (1.00 mL) was added HATU (41.8 mg, 110 µmol), DIPEA (105 µL, 600 µmol) and **3** (44.2 mg, 110 µmol) at 0 °C. The reaction mixture was stirred at room temperature for 19 h. The reaction was quenched by the addition of saturated aqueous NH_4Cl and the mixture was extracted with EtOAc. The organic layer was dried over $MgSO_4$ and concentrated under reduced pressure to obtain the crude amide. The crude amide and anisole (109 µL, 1.00 mmol) in CH_2Cl_2 (500 µL) were added 4 M HCl/dioxane (500 µL, 2.00 mmol) at 0 °C. The reaction mixture was stirred for 1 h at room temperature, and then concentrated under reduced pressure followed by purification with preparative RP-HPLC to obtain the *bis*-trifluoroacetate salt of the title compound **32** (19.0 mg, 24.9 µmol, 25% in 3 steps) as freeze-dried powder: t_R = 14.0 min (linear gradient of B in A, 20 to 50% over 30 min); ^1H NMR (500 MHz, MeOD): (mixture of diastereomers and rotamers) δ 1.45-3.29 (m, 15H), 3.36-4.56 (m, 12H), 6.96-7.67 (m, 10H, including indole C2~C6-H), 10.94 (br, 1H, indole N-H); $^{13}C\{^1H\}$ NMR (125 MHz, MeOD): (mixture of diastereomers and rotamers) δ 15.0, 30.5, 31.0, 31.5, 39.7, 41.5, 44.2, 44.9, 45.9, 47.2, 47.5, 53.4, 54.6, 65.4, 113.0, 119.2, 119.8, 120.7, 121.2, 123.6, 125.0, 125.2, 128.1, 129.8, 130.0, 131.0, 136.3, 138.5, 162.8, 173.9; HRMS (ESI), *m*/*z* calcd for $C_{30}H_{40}N_5O_2S$ $[M+H]^+$ 534.2897, found 534.2897. Anal. Calcd for $C_{30}H_{39}N_5O_2S$·2TFA: C, 53.61; H, 5.43; N, 9.19. Found: C, 51.74; H, 5.17; N, 9.05.

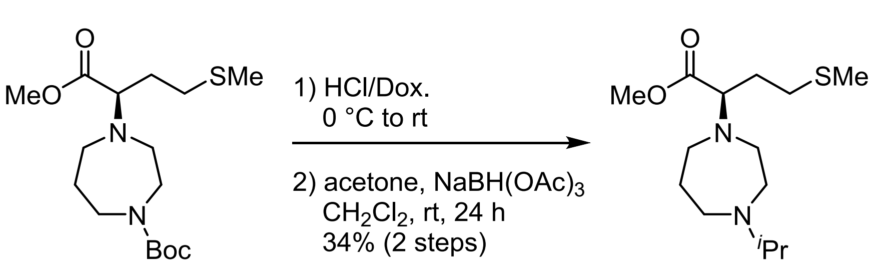

4 **20**

Methyl (*R*)-2-(4-isopropyl-1,4-diazepan-1-yl)-4-(methylthio)butanoate (**20**): The compound **4** (346 mg,

1.00 mmol) was added 4 M HCl/dioxane (5.00 mL, 20.0 mmol) at 0 °C. The reaction mixture was warmed to room temperature and stirred for 1 h. The reaction mixture was concentrated under reduced pressure to obtain the crude amine. The crude amine in CH_2Cl_2 (10.0 mL) was added acetone (346 µL, 4.70 mmol) and $NaBH(OAc)_3$ (636 mg, 3.00 mmol) at room temperature. The reaction mixture was stirred at room temperature for 24 h. The reaction quenched by the addition of saturated aqueous $NaHCO_3$ and the mixture was extracted with CH_2Cl_2. The organic layer was dried over $MgSO_4$ and concentrated under reduced pressure. The crude mixture was purified by automated silica gel flush column chromatography system (Isolera One) with $CHCl_3$/ MeOH (92:8 to 66:34) to obtain the title compound **20** (98.1 mg, 34% in 2 steps) as colorless oil: ^{1}H NMR (500 MHz, $CDCl_3$): δ 1.06 (d, *J* = 6.0 Hz, 6H), 1.83-2.02 (m, 5H), 2.10 (s, 3H), 2.57-2.61 (m, 2H), 2.70-2.79 (m, 5H), 2.87-2.93 (m, 2H), 3.04 (s, 1H), 3.45 (m, 1H), 3.69 (s, 3H); $^{13}C\{^1H\}$ NMR (125 MHz, $CDCl_3$): δ 15.6, 18.2, 18.3, 29.4, 31.2 (2C), 49.6, 50.6, 51.4, 52.4 (2C), 55.6, 66.3, 173.6; HRMS (ESI), *m*/*z* calcd for $C_{14}H_{29}N_2O_2S$ $[M+H]^+$ 289.1944, found 289.1940.

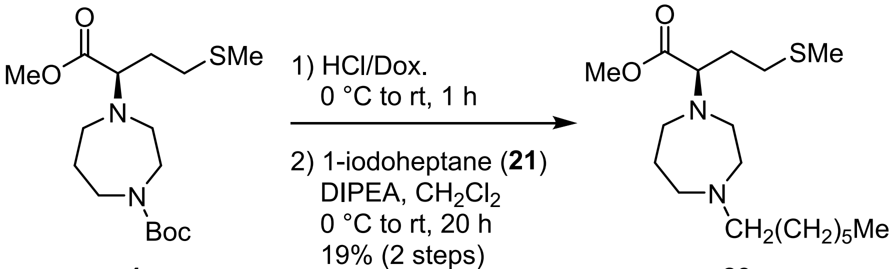

Methyl (*R*)-2-(4-heptyl-1,4-diazepan-1-yl)-4-(methylthio)butanoate (**23**): The compound **4** (346 mg, 1.00 mmol) were added 4 M HCl/dioxane (5.00 mL, 20.0 mmol) at 0 °C. The reaction mixture was stirred at room temperature for 1 h, and then concentrated under reduced pressure to obtain the crude amine. The crude amine in CH_2Cl_2 (5.00 mL) was added DIPEA (697 µL, 4.00 mmol), 1-iodoheptane **21** (330 µL, 2.00 mmol) at room temperature. The reaction mixture was stirred at room temperature for 20 h. The reaction was quenched by the addition of saturated aqueous NH_4Cl and the mixture was extracted with EtOAc. The organic layer was dried over $MgSO_4$ and concentrated under reduced pressure. The crude compound was purified by automated silica gel flush column chromatography system (Isolera One) with $CHCl_3$/ MeOH (99:1 to 90:10) to obtain the title compound **23** (66.4 mg, 19% in 2 steps) as a white solid: ^{1}H NMR (500 MHz, $CDCl_3$): δ 0.88 (t, *J* = 7.0 Hz, 3H), 1.18-1.35 (m, 9H), 1.86-2.01 (m, 5H), 2.10 (s, 3H), 2.56-2.61 (m, 4H), 2.71-2.98 (m, 8H), 3.46-3.49 (m, 1H), 3.69 (s, 3H); $^{13}C\{^1H\}$ NMR (125 MHz, $CDCl_3$): δ 14.2, 15.6, 22.7, 27.6, 29.7 (2C), 31.2 (2C), 31.9 (2C), 51.5 (2C), 53.8, 57.0 (2C), 58.1, 66.1, 173.6; HRMS (ESI), *m*/*z* calcd for $C_{18}H_{37}N_2O_2S$ $[M+H]^+$ 345.2570, found 345.2569.

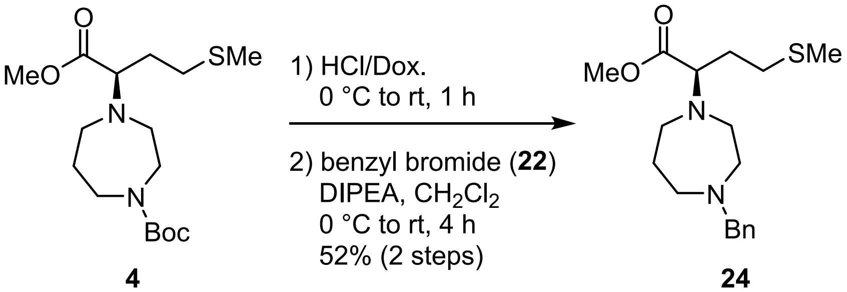

4 **24**

Methyl (*R*)-2-(4-benzyl-1,4-diazepan-1-yl)-4-(methylthio)butanoate (**24**): The compound **4** (173 mg, 0.500 mmol) was added 4 M HCl/dioxane (2.50 mL, 10.0 mmol) at 0 °C. The reaction mixture was stirred at room temperature for 1 h, and then concentrated under reduced pressure to obtain the crude amine. The crude amine in CH_2Cl_2 (2.5 mL) was added DIPEA (348 µL, 2.00 mmol) and benzyl bromide **22** (59.0 µL, 600 µmol) at room temperature. The reaction mixture was stirred at room temperature for 4 h. The reaction mixture was added silica gel and evaporated under reduced pressure. The residue was purified by automated silica gel flush column chromatography system (Isolera One) with *n*-hexane/AcOEt/ (80:20 to 20:80) to obtain the title compound **24** (260 mg, 52% in 2 steps) as colorless oil: ^{1}H NMR (500 MHz, $CDCl_3$): δ 1.77-1.80 (m, 2H), 1.86-2.01 (m, 2H), 2.10 (s, 3H), 2.59-2.78 (m, 8H), 2.89-2.94 (m, 2H), 3.45-3.48 (m, 1H), 3.65 (s, 2H), 3.69 (s, 3H), 7.22-7.34 (m, 5H); $^{13}C\{^{1}H\}$ NMR (125 MHz, $CDCl_3$): δ 15.6, 28.6, 29.4, 31.2, 50.8, 51.3, 51.5, 54.2, 57.1, 62.4, 66.2, 127.1, 128.3 (4C), 129.1, 173.8; HRMS (ESI), *m*/*z* calcd for $C_{18}H_{29}N_2O_2S$ $[M+H]^+$ 337.1944, found 337.1940.

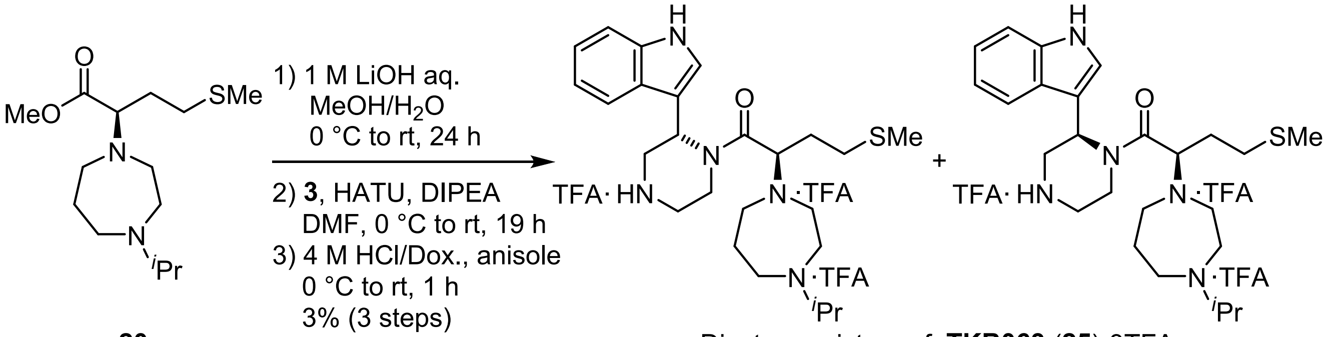

20

Diastereomixture of **TKB063** (**25**)·3TFA
(**TKB063A** (**25A**)·3TFA or **TKB063B** (**25B**)·3TFA)

(2*R*)-1-(2-(1*H*-Indol-3-yl)piperazin-1-yl)-2-(4-isopropyl-1,4-diazepan-1-yl)-4-(methylthio)butan-1-one·3TFA (**25**·3TFA, **TKB-063**·3TFA): To a solution of compound **20** (28.9 mg, 100 µmol) in MeOH (1.00 mL) was added 1.00 M LiOH aq. (250 µL, 250 µmol) at 0 °C. The reaction mixture was stirred at room temperature for 24 h. The reaction was quenched by the addition of saturated aqueous NH_4Cl and the mixture was extracted with $CHCl_3$. The organic layer was dried over $MgSO_4$ and concentrated under reduced pressure to obtain the crude carboxylic acid. The crude carboxylic acid in DMF (1.00 mL) was added HATU (41.8 mg, 110 µmol), DIPEA (105 µL, 600 µmol) and compound **3** (44.2 mg, 110 µmol) at 0 °C. The reaction mixture was

warmed to room temperature and stirred for 19 h. The reaction was quenched by the addition of saturated aqueous NH_4Cl and the mixture was extracted with EtOAc. The organic layer was dried over $MgSO_4$ and concentrated under reduced pressure to obtain the crude amide. The crude amide and anisole (109 μL, 1.00 mmol) in CH_2Cl_2 (500 μL) were added 4 M HCl/dioxane (500 μL, 2.00 mmol) at 0 °C. The reaction mixture was stirred at room temperature for 1 h, and then concentrated under reduced pressure followed by purification with preparative RP-HPLC to obtain the *tris*-trifluoroacetate salt of the title compound **25** (2.40 mg, 2.98 μmol, 3% in 3 steps) as freeze-dried powder:

TKB063A (**25A**); t_R = 23.0 min (linear gradient of B in A, 12 to 27% over 30 min); ^{1}H NMR (500 MHz, MeOD): (mixture of rotamers) δ 0.77-3.13 (m, 26H), 3.39-3.71 (m, 4H), 3.90-4.21 (m, 2H), 6.96-7.17 (m, 1H, indole C5,C6-H), 7.18-7.28 (m, 1H, indole C5,C6-H), 7.33-7.53 (m, 2H, indole C2-H and C4,C7-H), 7.54-7.65 (m, 1H, indole C4,C7-H); ^{13}C{^{1}H} NMR (125 MHz, MeOD): (mixture of rotamers) δ 15.4, 16.6, 25.3, 26.1, 30.8, 32.1, 32.3, 36.9, 39.6, 44.4, 53.0, 53.9, 60.2, 60.6, 64.4, 65.3, 113.4, 119.7, 121.1, 123.6, 124.9, 125.0, 126.5, 138.7, 162.3; HRMS (ESI), *m*/*z* calcd for $C_{25}H_{40}N_5OS$ $[M+H]^+$ 458.2948, found 458.2948. Anal. Calcd for $C_{25}H_{39}N_5OS\cdot 3TFA$: C, 46.56; H, 5.29; N, 8.76. Found: C, 46.10; H, 5.41; N, 8.19.

TKB063B (**25B**); t_R = 24.7 min (linear gradient of B in A, 12 to 27% over 30 min); ^{1}H NMR (500 MHz, MeOD): (mixture of rotamers) δ 0.77-3.16 (m, 25H), 3.36-3.71 (m, 5H), 3.81-4.16 (m, 2H), 7.07 (t, *J* = 7.4 Hz, 1H, indole C5,C6-H), 7.15-7.25 (m, 1H, indole C5,C6-H), 7.43 (d, *J* = 8.1 Hz, 1H, indole C2-H), 7.48-7.60 (m, 1H, indole C4,C7-H), 7.61-7.78 (m, 1H, indole C4,C7-H); ^{13}C{^{1}H} NMR (125 MHz, MeOD): (mixture of rotamers) δ 15.1, 17.2, 24.3, 26.6, 30.8, 32.1, 39.4, 44.7, 45.2, 46.5, 47.3, 50.7, 52.0, 52.6, 60.5, 63.9, 111.6, 113.0, 120.7, 123.6, 125.1, 125.2, 127.3, 138.6, 162.5; HRMS (ESI), *m*/*z* calcd for $C_{25}H_{40}N_5OS$ $[M+H]^+$ 458.2948, found 458.2946. Anal. Calcd for $C_{25}H_{39}N_5OS\cdot 3TFA$: C, 46.56; H, 5.29; N, 8.76. Found: C, 47.34; H, 5.49; N, 8.67.

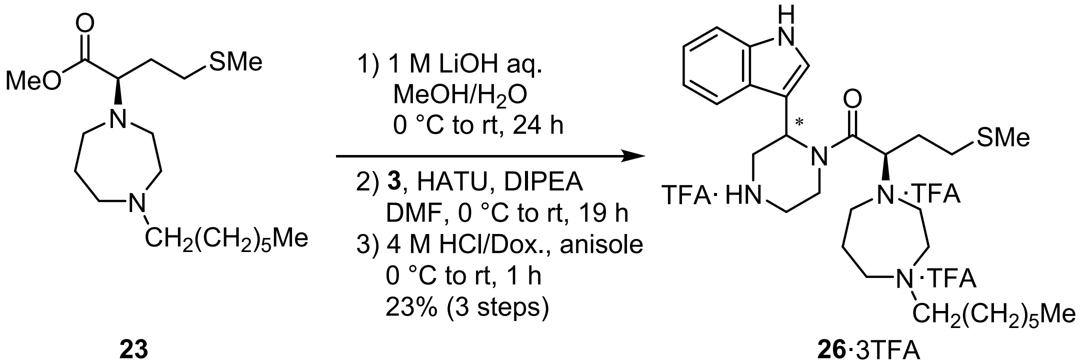

23 **26**·3TFA

(2*R*)-1-(2-(1*H*-Indol-3-yl)piperazin-1-yl)-2-(4-heptyl-1,4-diazepan-1-yl)-4-(methylthio)butan-1-one·3TFA (**26**·3TFA): To a solution of compound **23** (34.5 mg, 100 μmol) in MeOH (1.00 mL) was added 1.00 M LiOH aq. (250 μL, 0.250 mmol) at 0 °C. The reaction mixture was stirred at room temperature for 24 h. The reaction was quenched by the addition of saturated aqueous NH_4Cl and the mixture was extracted with $CHCl_3$. The organic layer was dried over $MgSO_4$ and concentrated under reduced pressure to obtain the crude carboxylic acid. The crude carboxylic acid in DMF (1.00 mL) was added HATU (41.8 mg, 110 μmol), DIPEA (105 μL, 600 μmol) and compound **3** (44.2 mg, 110 μmol) at 0 °C. The reaction mixture was stirred at room temperature

for 19 h. The reaction was quenched by the addition of saturated aqueous NH_4Cl and the mixture was extracted with EtOAc. The organic layer was dried over $MgSO_4$ and concentrated under reduced pressure to obtain the crude amide. The crude amide and anisole (109 μL, 1.00 mmol) in CH_2Cl_2 (500 μL) were added 4 M HCl/dioxane (500 μL, 2.00 mmol) at 0 °C. The reaction mixture was warmed to room temperature and stirred for 1 h. The reaction mixturte was concentrated under reduced pressure followed by purification with preparative RP-HPLC to obtain the *tris*-trifluoroacetate salt of the title compound **26** (22.8 mg, 22.8 μmol, 23% in 3 steps) as freeze-dried powder: t_R = 19.7 min and 20.6 min (linear gradient of B in A, 25 to 45% over 40 min); ^{1}H NMR (500 MHz, MeOD): (mixture of diastereomers and rotamers) δ 0.93 (t, J = 13.5 Hz, 3H), 1.05-1.48 (m, 10H), 1.50-3.28 (m, 20H), 3.34-4.24 (m, 7H), 7.07 (t, J = 7.2 Hz, 1H, indole C5,C6-H), 7.18 (t, J = 7.6 Hz, 1H, indole C5,C6-H), 7.30-7.72 (m, 3H, indole C2-H and C4,C7-H), 10.95 (br, 1H, indole N-H); ^{13}C{^{1}H} NMR (125 MHz, MeOD): (mixture of diastereomers and rotamers) δ 14.4, 15.3, 23.6, 25.4, 26.0, 27.5, 29.9, 32.1, 32.7, 36.9, 39.5, 44.5, 45.2, 46.7, 52.0, 52.9, 54.2, 57.4, 58.8, 63.8, 112.9, 117.1, 119.8, 120.6, 123.5, 125.1, 126.5, 138.6, 163.3; HRMS (ESI), m/z calcd for $C_{29}H_{48}N_5OS$ $[M+H]^+$ 514.3574, found 514.3571. Anal. Calcd for $C_{29}H_{47}N_5OS·3TFA$: C, 49.12; H, 5.89; N, 8.18. Found: C, 49.35; H, 6.26; N, 8.71.

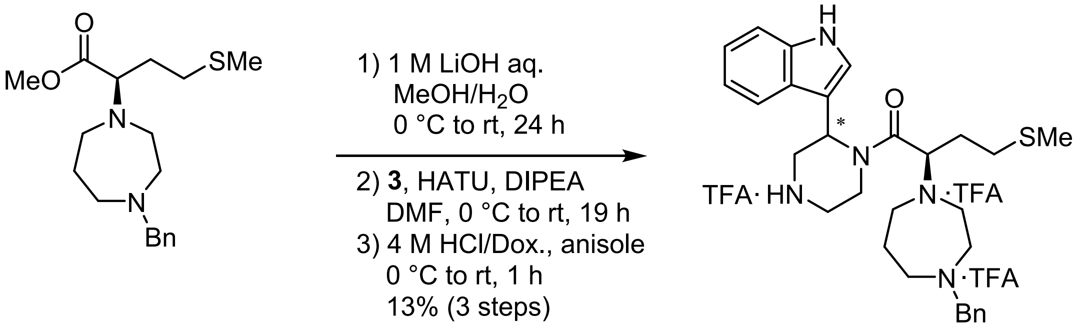

24 **27**·3TFA

(2*R*)-1-(2-(1*H*-Indol-3-yl)piperazin-1-yl)-2-(4-benzyl-1,4-diazepan-1-yl)-4-(methylthio)butan-1-one·3TFA (**27**·3TFA): To a solution of compound **24** (33.6 mg, 100 μmol) in MeOH (1.00 mL) was added 1.00 M LiOH aq. (250 μL, 250 μmol) at 0 °C. The reaction mixture was stirred at room temperature for 24 h. The reaction was quenched by the addition of saturated aqueous NH_4Cl and the mixture was extracted with $CHCl_3$. The organic layer was dried over $MgSO_4$ and concentrated under reduced pressure to obtain the crude carboxylic acid. The crude carboxylic acid in DMF (1.00 mL) was added HATU (41.8 mg, 110 μmol), DIPEA (105 μL, 600 μmol) and compound **3** (44.2 mg, 110 μmol) at 0 °C. The reaction mixture was stirred at room temperature for 19 h. The reaction was quenched by the addition of saturated aqueous NH_4Cl and the mixture was extracted with EtOAc. The organic layer was dried over $MgSO_4$ and concentrated under reduced pressure to obtain the crude amide. The crude amide and anisole (109 μL, 1.00 mmol) in CH_2Cl_2 (500 μL) were added 4 M HCl/dioxane (500 μL, 2.00 mmol) at 0 °C. The reaction mixture was stirred at room temperature for 1 h, and then concentrated under reduced pressure followed by purification with preparative RP-HPLC to obtain the *tris*-trifluoroacetate salt of the title compound **27** (10.9 mg, 12.9 μmol, 13% in 3 steps) as freeze-dried powder: t_R = 12.9 min and 13.3 min (linear gradient of B in A, 20 to 50% over 30 min); ^{1}H NMR (500 MHz, MeOD): (mixture

of diastereomers and rotamers) δ 1.23-3.28 (m, 17H), 3.34-4.53 (m, 10H), 6.94-7.67 (m, 10H, including indole C2~C6-H), 10.95 (br, 1H, indole N-H); $^{13}C\{^{1}H\}$ NMR (125 MHz, MeOD): (mixture of diastereomers and rotamers) δ 15.1, 15.3, 25.4, 25.8, 32.1, 36.9, 39.5, 44.5, 45.9, 45.1, 46.5, 51.6, 52.5, 53.8, 57.3, 57.5, 61.8, 63.7, 71.4, 110.6, 111.8, 113.0, 119.9, 120.7, 123.6, 125.0, 126.6, 126.9, 130.4, 131.2, 132.0, 132.2, 138.6, 163.0; HRMS (ESI), *m*/*z* calcd for $C_{29}H_{40}N_5OS$ $[M+H]^+$ 506.2948, found 506.2949. Anal. Calcd for $C_{29}H_{39}N_5OS \cdot 3TFA$: C, 49.59; H, 4.99; N, 8.26. Found: C, 48.76; H, 5.04; N, 7.79.

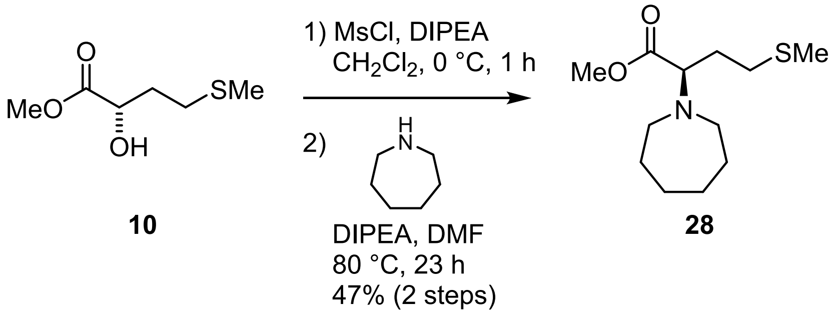

Methyl (*R*)-2-(azepan-1-yl)-4-(methylthio)butanoate (**28**): To a solution of compound **10** (328 mg, 2.00 mmol) and DIPEA (1.05 mL, 6.00 mmol) in CH_2Cl_2 (10.0 mL) was added MsCl (263 μL, 3.00 mmol) at 0 °C. The reaction mixture was stirred at room temperature for 1 h. The reaction was quenched by the addition of saturated aqueous NH_4Cl and the mixture was extracted with CH_2Cl_2. The organic layer was dried over $MgSO_4$ and concentrated under reduced pressure to obtain the crude mesylate. The crude mesylate and DIPEA (1.05 mL, 6.00 mmol) in DMF (4.00 mL) was added hexamethyleneimine (451 μL, 4.00 mmol) at room temperature. The reaction mixture was stirred at 80 °C for 23 h. The reaction was quenched by the addition of saturated aqueous NH_4Cl and the mixture was extracted with EtOAc. The organic layer was dried over $MgSO_4$ and concentrated under reduced pressure followed by purification using automated silica gel flush column chromatography system (Isolera One) with *n*-hexane/AcOEt (92:8 to 90:10) to obtain the title compound **28** (231 mg, 47% in 2 steps) as colorless oil: ^{1}H NMR (500 MHz, $CDCl_3$): δ 1.57-1.63 (m, 8H), 1.88-1.98 (m, 2H), 2.11 (s, 3H), 2.58-2.67 (m, 4H), 2.80-2.84 (m, 2H), 3.43-3.46 (m, 1H), 3.68 (s, 3H); $^{13}C\{^{1}H\}$ NMR (125 MHz, $CDCl_3$): δ 15.6, 27.2 (2C), 29.7, 30.0 (2C), 31.2, 51.2, 52.1 (2C), 66.7, 174.1; HRMS (ESI), *m*/*z* calcd for $C_{12}H_{24}NO_2S$ $[M+H]^+$ 246.1522, found 246.1526.

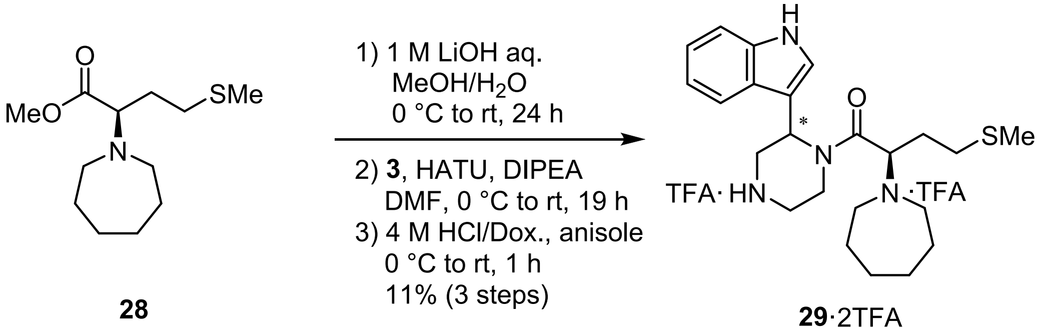

28 **29**·2TFA

(2*R*)-1-(2-(1*H*-Indol-3-yl)piperazin-1-yl)-2-(azepan-1-yl)-4-(methylthio)butan-1-one·2TFA (**29**·2TFA): To a solution of compound **28** (24.5 mg, 100 μmol) in MeOH (1.00 mL) was added 1.00 M LiOH aq. (250 μL, 250 μmol) at 0 °C. The reaction mixture was stirred at room temperature for 24 h. The reaction was quenched by the addition of saturated aqueous NH_4Cl and the mixture was extracted with $CHCl_3$. The organic layer was dried over $MgSO_4$ and concentrated under reduced pressure to obtain the crude carboxylic acid. The crude carboxylic acid in DMF (1.00 mL) was added HATU (41.8 mg, 110 μmol), DIPEA (105 μL, 600 μmol) and compound **3** (44.2 mg, 110 μmol) at 0 °C. The reaction mixture was stirred at room temperature for 19 h. The reaction was quenched by the addition of saturated aqueous NH_4Cl and the mixture was extracted with EtOAc. The organic layer was dried over $MgSO_4$ and concentrated under reduced pressure to obtain the crude amide. The crude amide and anisole (109 μL, 1.00 mmol) in CH_2Cl_2 (500 μL) were added 4 M HCl/dioxane (500 μL, 2.00 mmol) at 0 °C. The reaction mixture was stirred for 1 h at room temperature, and then concentrated under reduced pressure followed by purification with preparative RP-HPLC to obtain the *bis*-trifluoroacetate salt of the title compound **29** (7.10 mg, 11.0 μmol, 11% in 3 steps) as freeze dried powder: t_R = 17.4 min and 18.5 min (linear gradient of B in A, 15 to 35% over 40 min); ^{1}H NMR (500 MHz, MeOD): (mixture of diastereomers and rotamers) δ 0.81-3.25 (m, 19H), 3.40-4.81 (m, 8H), 7.02-7.15 (m, 1H, indole C5,C6-H), 7.16-7.28 (m, 1H, indole C5,C6-H), 7.37-7.52 (m, 1H, indole C2-H), 7.53-7.70 (m, 2H, indole C4,C7-H); ^{13}C{^{1}H} NMR (125 MHz, MeOD): (mixture of diastereomers and rotamers) δ 14.9, 25.0, 25.1, 27.4, 28.0, 29.4, 37.5, 39.8, 43.7, 44.8, 45.9, 46.3, 47.4, 65.3, 110.4, 112.9, 123.7, 123.9, 125.0, 125.1, 126.9, 138.3, 167.7; HRMS (ESI), *m*/*z* calcd for $C_{23}H_{35}N_4OS$ $[M+H]^+$ 415.2526, found 415.2529. Anal. Calcd for $C_{23}H_{34}N_4OS$·2TFA: C, 50.46; H, 5.65; N, 8.72. Found: C, 46.70; H, 5.14; N, 7.47.

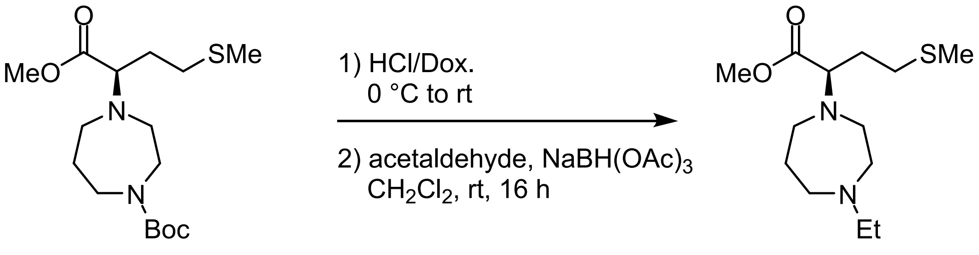

4 **33**

Methyl (*R*)-2-(4-ethyl-1,4-diazepan-1-yl)-4-(methylthio)butanoate (**33**): The compound **4** (193 mg, 557 μmol) was added 4 M HCl/dioxane (2.75 mL, 11 mmol) at 0 °C. The reaction mixture was stirred at room temperature for 3 h, and then concentrated under reduced pressure to obtain the crude amine. The crude amine in CH_2Cl_2 (5.50 mL) was added acetaldehyde (147 μL, 2.59 mmol) and $NaBH(OAc)_3$ (350 mg, 1.65 mmol) at room temperature. The reaction mixture was stirred at room temperature for 16 h. The reaction was quenched by the addition of saturated aqueous $NaHCO_3$ and the mixture was extracted with CH_2Cl_2. The organic layer was dried over $MgSO_4$ and concentrated under reduced pressure. The crude compound **33** was used immediately in next step without purification.

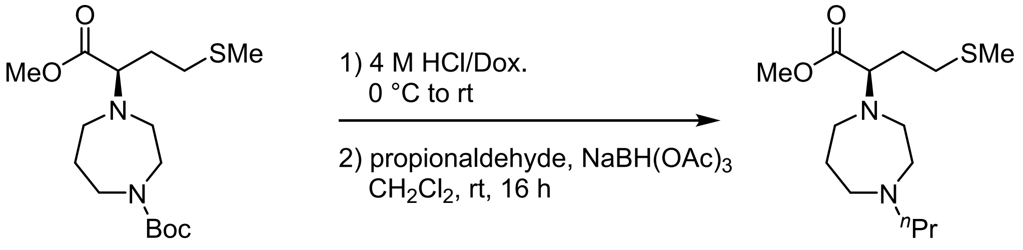

4 **34**

Methyl (*R*)-4-(methylthio)-2-(4-propyl-1,4-diazepan-1-yl)butanoate (**34**): The compound **4** (122 mg, 352 μmol) was added 4 M HCl/dioxane (721 μL, 2.88 mmol) at 0 °C. The reaction mixture was stirred at room temperature for 3 h, and then concentrated under reduced pressure to obtain the crude amine. The crude amine in CH_2Cl_2 (3.52 mL) was added propionaldehyde (120 μL, 677 μmol) and $NaBH(OAc)_3$ (223.8 mg, 432 μmol) at room temperature. The reaction mixture was stirred at room temperature for 16 h. The reaction was quenched by the addition of saturated aqueous $NaHCO_3$ and the mixture was extracted with CH_2Cl_2. The organic layer was dried over $MgSO_4$ and concentrated under reduced pressure. The crude compound **34** was used immediately in next step without purification.

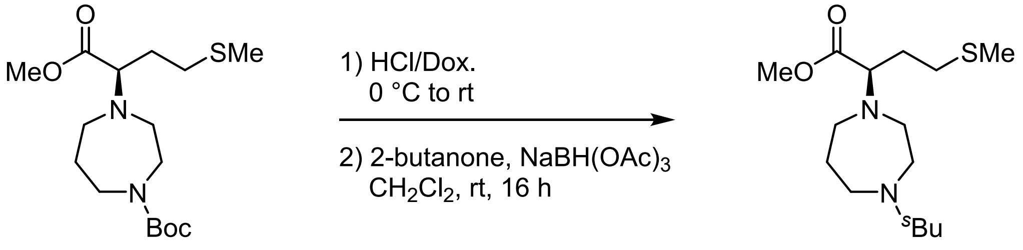

4 **35**

Methyl (2*R*)-2-(4-(*sec*-butyl)-1,4-diazepan-1-yl)-4-(methylthio)butanoate (**35**): The compound **4** (194 mg, 560 μmol) was added 4 M HCl/dioxane (11.2 mL, 2.80 mmol) at 0 °C. The reaction mixture was stirred at room temperature for 3 h, and then concentrated under reduced pressure to obtain the crude amine. The crude amine in CH_2Cl_2 (5.60 mL) was added 2-butanone (236 μL, 2.63 mmol) and $NaBH(OAc)_3$ (356 mg, 1.68 mmol) at room temperature. The reaction mixture was stirred at room temperature for 17 h. The reaction was quenched by the addition of saturated aqueous $NaHCO_3$ and the mixture was extracted with CH_2Cl_2. The organic layer was dried over $MgSO_4$ and concentrated under reduced pressure. The crude compound **35** was used immediately in next step without purification.

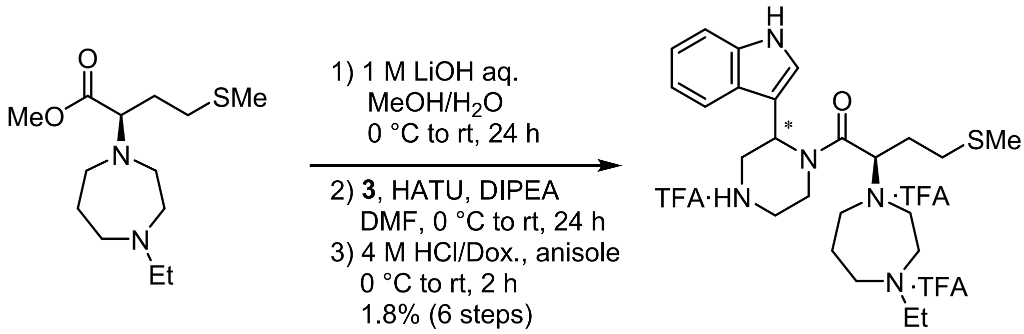

33 **TON01** (**36**)·3TFA

(2*R*)-1-(2-(1*H*-Indol-3-yl)piperazin-1-yl)-2-(4-benzyl-1,4-diazepan-1-yl)-4-(methylthio)butan-1-one·3TFA (**36**·3TFA, **TON01**·3TFA): To a solution of compound **33** (84 mg, 100 μmol) in MeOH (3.00 mL) were added 1.00 M LiOH aq. (760 μL, 0.765 mmol) at 0 °C. The reaction mixture was stirred at room temperature for 24 h. The reaction was quenched by the addition of saturated aqueous NH_4Cl and the mixture was extracted with $CHCl_3$. The organic layer was dried over $MgSO_4$ and concentrated under reduced pressure to obtain the crude carboxylic acid. The crude carboxylic acid in DMF (1.40 mL) was added HATU (53.2 mg, 0.139 mmol), DIPEA (133 μL, 761 μmol) and compound **3** (56.2 mg, 139 μmol) at 0 °C. The reaction mixture was stirred at room temperature for 24 h. The reaction was quenched by the addition of saturated aqueous NH_4Cl and the mixture was extracted with EtOAc. The organic layer was dried over $MgSO_4$ and concentrated under reduced pressure to obtain the crude amide. The crude amide and anisole (228 μL, 1.27 mmol) in CH_2Cl_2 (635 μL) were added 4 M HCl/dioxane (635 μL, 2.54 mmol) at 0 °C. The reaction mixture was stirred for 2 h at room temperature, and then concentrated under reduced pressure followed by purification with preparative RP-HPLC to obtain the *tris*-trifluoroacetate salt of the title compound **36** (8.02 mg, 10.2 μmol, 1.8% in 5 steps) as freeze-dried powder: t_R = 19.2 min and 20.2 min (linear gradient of B in A, 17 to 37% over 40 min); ^{1}H NMR (500 MHz, MeOD): (mixture of diastereomers and rotamers) δ 0.81-1.47 (m, 3H), 1.56-2.40 (m, 6H), 2.41-2.73 (m, 2H), 2.74-3.71 (m, 17H), 3.87-4.26 (m, 2H), 7.07 (t, J = 7.2 Hz, 1H, indole C5,C6-H), 7.19 (t, J = 7.7 Hz, 1H, indole C5,C6-H), 7.31-7.72 (m, 3H, indole C2-H and C4,C7-H), 10.9 (br, 1H, indole N-H); ^{13}C{^{1}H} NMR (125 MHz, MeOD): (mixture of diastereomers and rotamers) δ 8.49, 13.7, 13.9, 24.6, 24.9, 30.6, 35.5, 38.0, 43.6, 45.3, 50.6, 52.4, 55.3, 60.1, 62.4, 111.5, 117.3, 118.3, 119.2, 122.1, 123.6, 125.1, 137.2, 161.2; HRMS (ESI), m/z calcd for $C_{24}H_{38}N_5OS$ $[M+H]^+$ 444.2792, found 444.2796.

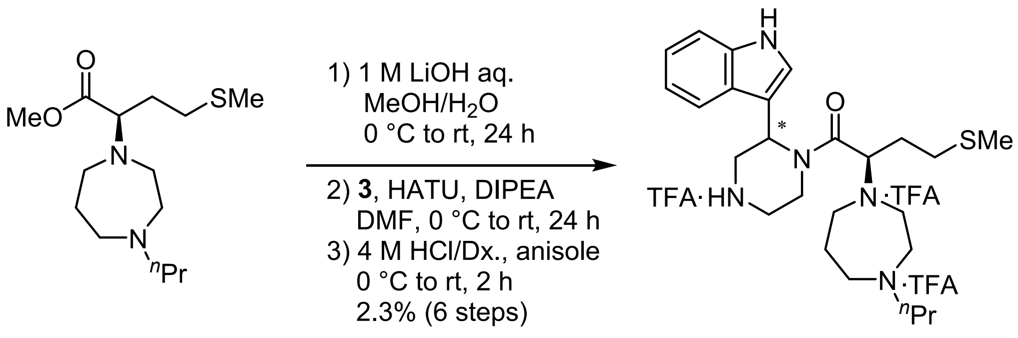

34 **TON02** (**37**)·3TFA

(2*R*)-1-(2-(1*H*-Indol-3-yl)piperazin-1-yl)-4-(methylthio)-2-(4-propyl-1,4-diazepan-1-yl)butan-1-one·3TFA (**37**·3TFA, **TON02**·3TFA): To a solution of compound **34** (71.3 mg, 247 μmol) in MeOH (2.50 mL) was added 1.00 M LiOH aq. (618 μL, 618 μmol) at 0 °C. The reaction mixture was stirred at room temperature for 24 h. The reaction was quenched by the addition of saturated aqueous NH_4Cl and the mixture was extracted with $CHCl_3$. The organic layer was dried over $MgSO_4$ and concentrated under reduced pressure to obtain the crude carboxylic acid. The crude carboxylic acid in DMF (1.40 mL) was added HATU (152.5 mg, 401 μmol), DIPEA (382 μL, 2.19 mmol) and compound **3** (161.0 mg, 401 μmol) at 0 °C. The reaction mixture was stirred at room temperature for 17 h. The reaction was quenched by the addition of saturated aqueous NH_4Cl and the mixture was extracted with EtOAc. The organic layer was dried over $MgSO_4$ and concentrated under reduced pressure to obtain the crude amide. The crude amide and anisole (397 μL, 3.65 mmol) in CH_2Cl_2 (3.65 mL) was added 4 M HCl/dioxane (7.32 mL, 2.54 mmol) at 0 °C. The reaction mixture was stirred at room temperature for 2 h, and then concentrated under reduced pressure followed by purification with preparative RP-HPLC to obtain the *tris*-trifluoroacetate salt of the title compound **37** (6.52 mg, 8.15 μmol, 2.3% in 5 steps) as freeze-dried powder: t_R = 21.4 min and 22.6 min (linear gradient of B in A, 10 to 70% over 60 min); ^{1}H NMR 500 MHz, MeOD): (mixture of diastereomers and rotamers) δ 0.72-1.15 (m, 2H), 1.16-1.52 (m, 1H), 1.55-2.37 (m, 10H), 2.41-2.76 (m, 3H), 2.77-3.30 (m, 8H), 3.37-3.78 (m, 6H), 3.89-4.36 (m, 3H), 6.97-7.14 (m, 1H, indole C5,C6-H), 7.15-7.30 (m, 1H, indole C5,C6-H), 7.33-7.76 (m, 3H, indole C2-H and C4,C7-H); ^{13}C{^{1}H} NMR (125 MHz, MeOD): (mixture of diastereomers and rotamers) δ 9.59, 13.8, 17.5, 24.1, 30.5, 35.6, 38.2, 43.1, 43.7, 45.8, 49.4, 50.1, 52.9, 55.5, 58.8, 62.9, 111.5, 114.7, 116.7, 118.3, 119.2, 122.1, 123.6, 137.1, 159.8; HRMS (ESI), *m*/*z* calcd for $C_{25}H_{40}N_5OS$ $[M+H]^+$ 458.2948, found 458.2946.

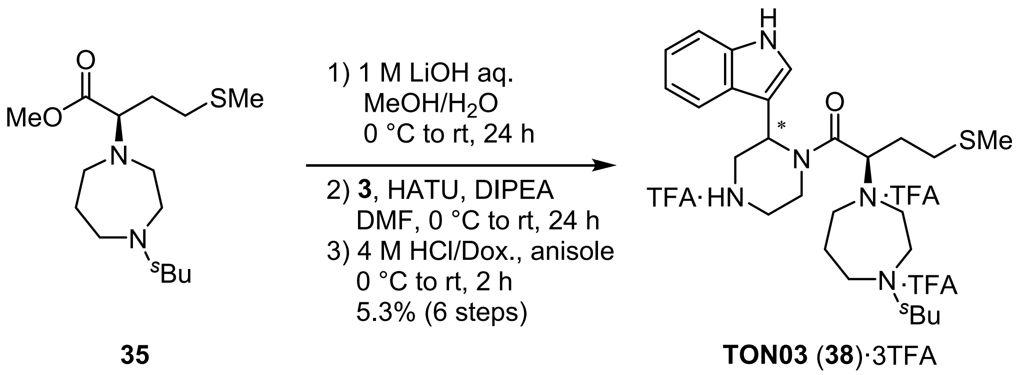

(2*R*)-1-(2-(1*H*-Indol-3-yl)piperazin-1-yl)-2-(4-(*sec*-butyl)-1,4-diazepan-1-yl)-4-(methylthio)butan-1-one·3TFA (**38**·3TFA, **TON03**·3TFA): To a solution of compound **35** (70.0 mg, 232 μmol) in MeOH (2.30 mL) was added 1.00 M LiOH aq. (579 μL, 579 μmol) at 0 °C. The reaction mixture was stirred at room temperature for 24 h. The reaction was quenched by the addition of saturated aqueous NH_4Cl and the mixture was extracted with $CHCl_3$. The organic layer was dried over $MgSO_4$ and concentrated under reduced pressure to obtain the crude carboxylic acid. The crude carboxylic acid in DMF (1.00 mL) was added HATU (79.1 mg, 208 μmol), DIPEA (181 μL, 1.04 mmol) and compound **3** (83.5 mg, 208 μmol) at 0 °C. The reaction mixture was stirred at room temperature for 24 h. The reaction was quenched by the addition of saturated aqueous NH_4Cl and the mixture was extracted with EtOAc. The organic layer was dried over $MgSO_4$ and concentrated under reduced pressure to obtain the crude amide. The crude amide and anisole (188 μL, 1.73 mmol) in CH_2Cl_2 (865 μL) were added 4 M HCl/dioxane (865 μL, 3.46 mmol) at 0 °C. The reaction mixture was stirred at room temperature for 2 h, and then concentrated under reduced pressure followed by purification with preparative RP-HPLC to obtain the *tris*-trifluoroacetate salt of the title compound **38** (23.9 mg, 29.4 μmol, 5.3% in 5 steps) as freeze dried powder: t_R = 23.7 min, 24.9 min (linear gradient of B in A, 17 to 37% over 40 min); ^{1}H NMR (400 MHz, MeOD): (mixture of diastereomers and rotamers) δ 0.57-3.27 (m, 27H), 3.32-3.92 (m, 5H), 3.99-4.23 (m, 2H), 7.06 (t, J = 7.6 Hz, 1H, indole C5,C6-H), 7.19 (t, J = 7.3, 1H, indole C5,C6-H), 7.43 (d, J = 8.2 Hz, 1H, indole C2-H), 7.49-7.59 (m, 1H, indole C4,C7-H), 7.50-7.73 (m, 1H, indole C4,C7-H); ^{13}C{^{1}H} NMR (125 MHz, MeOD): (mixture of diastereomers and rotamers) δ 10.8, 15.4, 24.2, 25.4, 25.8, 26.6, 32.2, 37.0, 39.5, 44.7, 45.4, 46.5, 47.7, 53.4, 55.3, 64.2, 66.5, 113.1, 116.5, 118.8, 120.9, 123.8, 125.3, 127.5, 138.9, 161.9; HRMS (ESI), m/z calcd for $C_{26}H_{42}N_5OS$ $[M+H]^+$ 472.3105, found 472.3106.

III. References

[1]Fredrich, S.; Bonasera, A.; Valderrey, V.; Hecht, S. Sensitive Assays by Nucleophile-Induced Rearrangement of Photoactivated Diarylethenes. *J. Am. Chem. Soc.* **2018**, *140*, 6432−6440.

[2]Luescher, M. U.; Vo, C.-V. T.; Bode, J. W. SnAP Reagents for the Synthesis of Piperazines and Morpholines.

Org. Lett. **2014**, *16*, 1236−1239.

[3]Griesbeck, A. G.; Heckroth, H. Stereoselective synthesis of 2-aminocyclobutanols via photocyclization of α-amido alkylaryl ketones: Mechanistic implications for the Norrish/Yang reaction. *J. Am. Chem. Soc.* **2002**, *124*, 396−403.

IV. ^{1}H NMR and ^{13}C NMR charts (compounds 25A, 25B and 38)

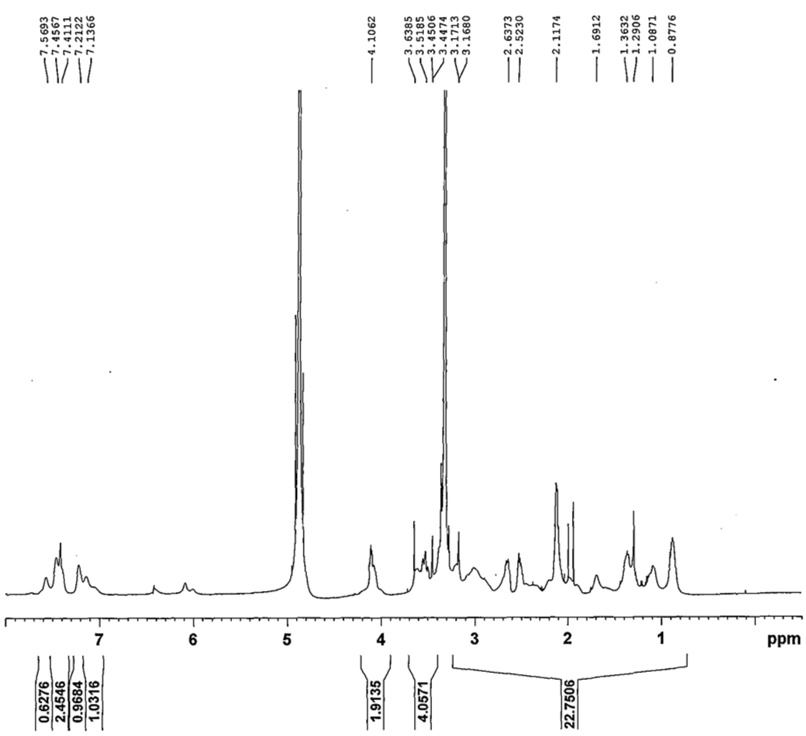

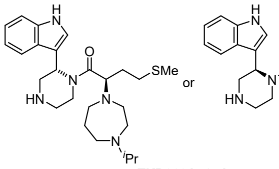

TKB063A (25A)

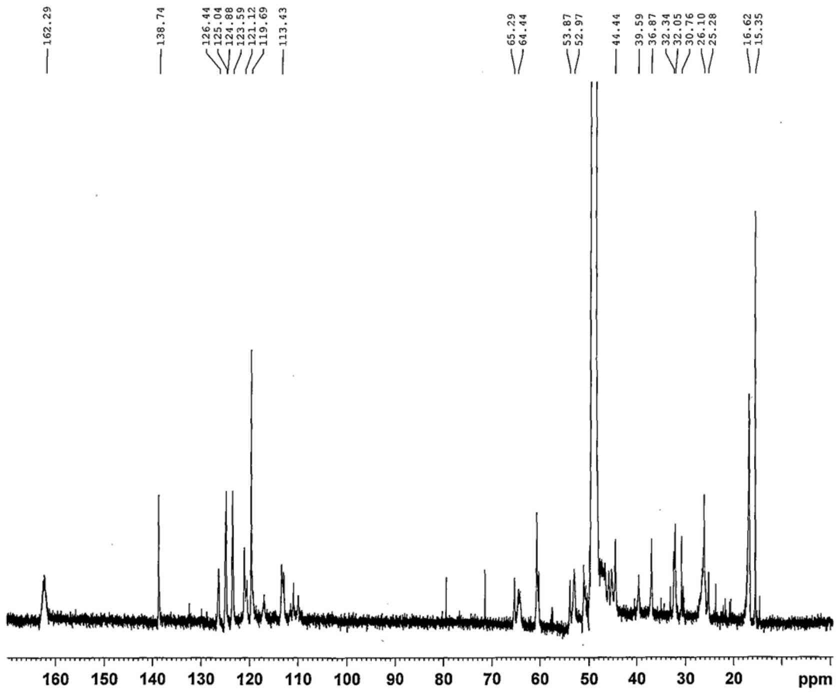

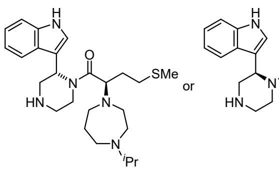

TKB063A (25A)

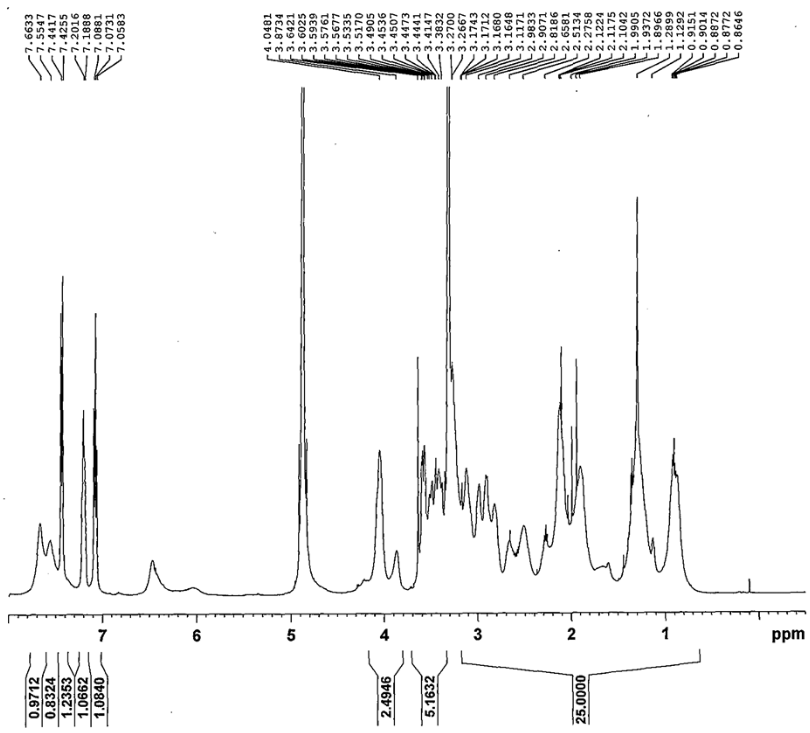

NAME	MKU-1614
EXPNO	4
PROCNO	1
Date_	20191126
Time	16.00
INSTRUM	av500
PROBHD	5 mm CPDCH 13C
PULPROG	zg30
TD	65536
SOLVENT	MeOD
NS	376
DS	2
SWH	10330.578 Hz
FIDRES	0.157632 Hz
AQ	3.1720407 se
RG	25.4
DW	48.400 us
DE	6.00 us
TE	298.0 K
D1	1.00000000 se
TD0	1
======== CHANNEL f1 ======	
NUC1	1H
P1	10.00 us
PL1	-4.00 dB
PL1W	6.30957365 W
SFO1	500.1330885 MH
SI	32768
SF	500.1292387 MH
WDW	EM
SSB	0
LB	0.30 Hz
GB	0
PC	0.50

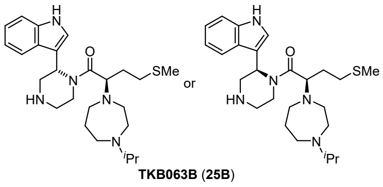

TKB063B (25B)

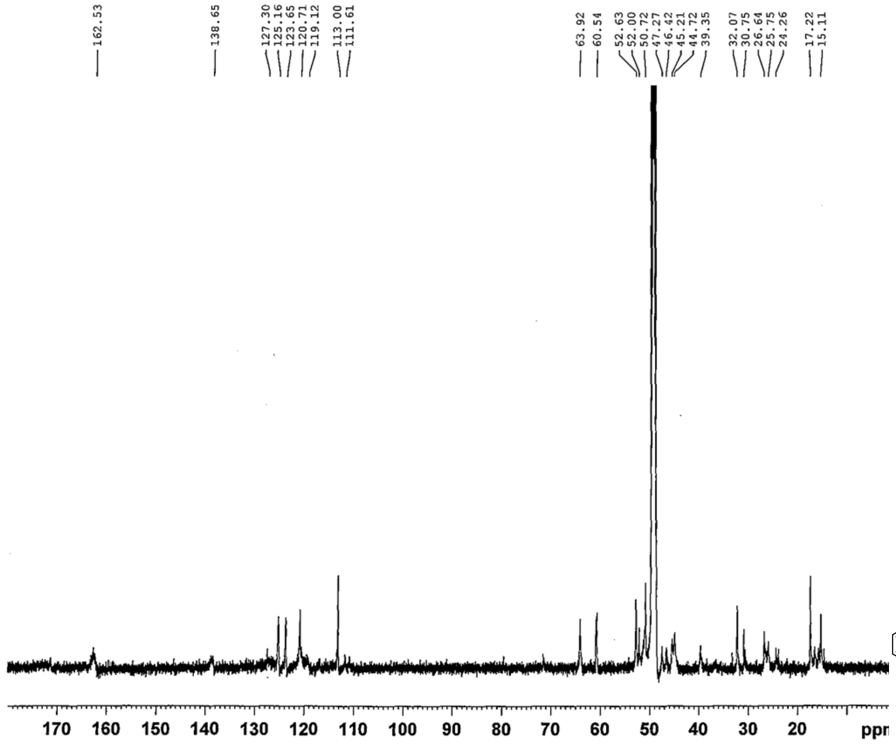

IBB-nmr Analysis

NAME	MKU-1614
EXPNO	6
PROCNO	1
Date_	20191127
Time	11.59
INSTRUM	av500
PROBHD	5 mm CPDCH 13C
PULPROG	zgpg30
TD	65536
SOLVENT	MeOD
NS	921
DS	4
SWH	30030.029 Hz
FIDRES	0.458222 Hz
AQ	1.0912410 sec
RG	228.1
DW	16.650 usec
DE	20.00 usec
TE	298.1 K
D1	2.00000000 sec
D11	0.03000000 sec
TD0	1
======== CHANNEL f1 ========	
NUC1	13C
P1	10.00 usec
PL1	-5.00 dB
PL1W	14.29790783 W
SFO1	125.7703643 MHz
======== CHANNEL f2 ========	
CPDPRG2	waltz16
NUC2	1H
PCPD2	80.00 usec
PL2	-4.00 dB
PL12	14.06 dB
PL13	17.00 dB
PL2W	6.30957365 W
PL12W	0.09862794 W
PL13W	0.05011872 W
SFO2	500.1320005 MHz
SI	32768
SF	125.7576125 MHz
WDW	EM
SSB	0
[illegible]	1.00 Hz

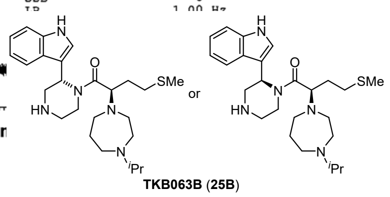

TKB063B (25B)

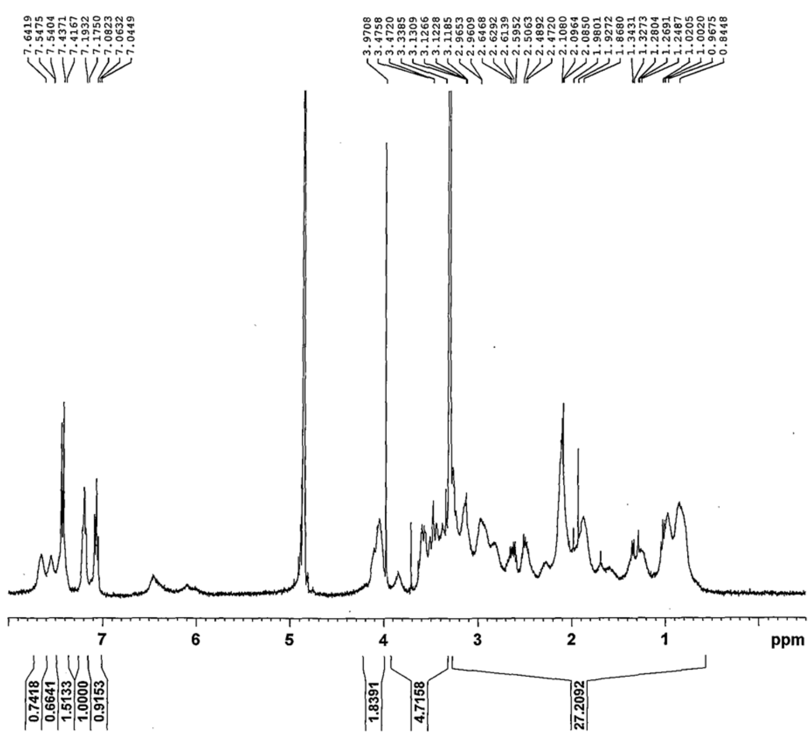


```
NAME            TON-0233
EXPNO                  1
PROCNO                 1
Date_           20210113
Time               16.04
INSTRUM            av400
PROBHD   5 mm PABBO BB-
PULPROG             zg30
TD                 65536
SOLVENT             MeOD
NS                    16
DS                     2
SWH          8223.685 Hz
FIDRES       0.125483 Hz
AQ          3.9846387 se
RG                   203
DW             60.800 us
DE               6.50 us
TE              297.7 K
D1        1.00000000 se
TD0                    1

======== CHANNEL f1 ======
NUC1                  1H
P1              14.00 us
PL1             -1.80 dB
PL1W       14.82738590 W
SFO1      400.1324710 MH
SI                 32768
SF        400.1300117 MH
WDW                   EM
SSB                    0
LB               0.30 Hz
GB                     0
PC                  1.00
```

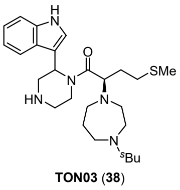

TON03 (38)

IBB-nmr Analysis

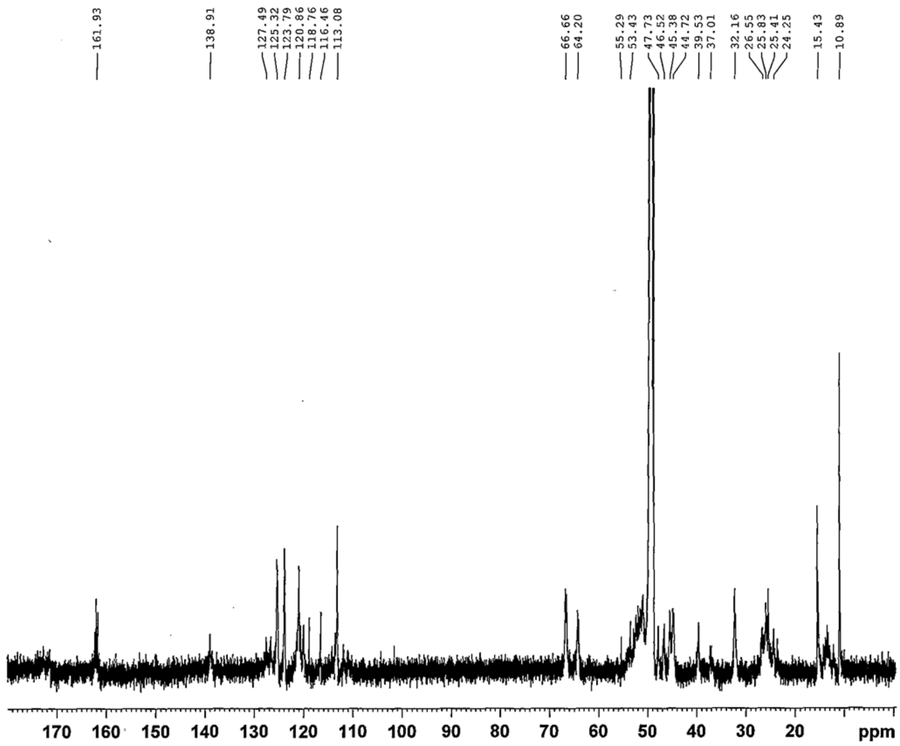


```
NAME       TON-0232 carbon
EXPNO                    1
PROCNO                   1
Date_             20210825
Time                 20.09
INSTRUM              av500
PROBHD     5 mm CPDCH 13C
PULPROG            zgpg30
TD                   65536
SOLVENT               MeOD
NS                    1024
DS                       4
SWH           30030.029 Hz
FIDRES         0.458222 Hz
AQ           1.0912410 sec
RG                   456.1
DW             16.650 usec
DE              20.00 usec
TE                 298.0 K
D1          2.00000000 sec
D11         0.03000000 sec
TD0                      1

======== CHANNEL f1 ========
NUC1                   13C
P1              10.00 usec
PL1               -5.00 dB
PL1W         14.29790783 W
SFO1        125.7703643 MHz

======== CHANNEL f2 ========
CPDPRG2            waltz16
NUC2                    1H
PCPD2           80.00 usec
PL2               -4.00 dB
PL12              14.06 dB
PL13              17.00 dB
PL2W          6.30957365 W
PL12W         0.09862794 W
PL13W         0.05011872 W
SFO2        500.1320005 MHz
SI                   32768
SF          125.7575956 MHz
WDW                     EM
SSB                      0
LB                 1.00 Hz
                         0
                         0
```

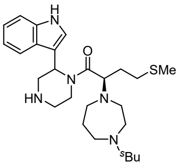

TON03 (38)

V. High Resolution Mass Spectrometry (HRMS) charts (compounds 25A, 25B and 38)

Mass Spectrum SmartFormula Report

**Analysis Info**			
Analysis Name	D:\Data\B\mkurakami\20200114\20200114000004.d	Acquisition Date	1/14/2020 2:31:27 PM
Method	esi_pos_low.m	Operator	BDAL@DE
Sample Name	MKU-1614	Instrument	micrOTOF 000000.00000
Comment	(+)-TKB063 HRMS		

Acquisition Parameter

Source Type	ESI	Ion Polarity	Positive	Set Nebulizer	0.4 Bar
Focus	Not active			Set Dry Heater	200 °C
Scan Begin	50 m/z	Set Capillary	4500 V	Set Dry Gas	4.0 l/min
Scan End	3000 m/z	Set End Plate Offset	-500 V	Set Divert Valve	Source

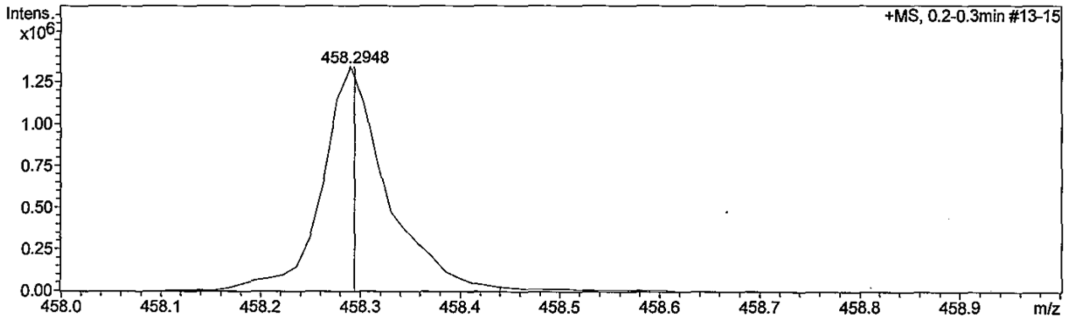

Meas. m/z	#	Ion Formula	m/z	err [ppm]	mSigma	# mSigma	Score	rdb	e⁻ Conf	N-Rule
458.2948	1	C25H40N5OS	458.2948	0.0	2.7	1	100.00	8.5	even	ok

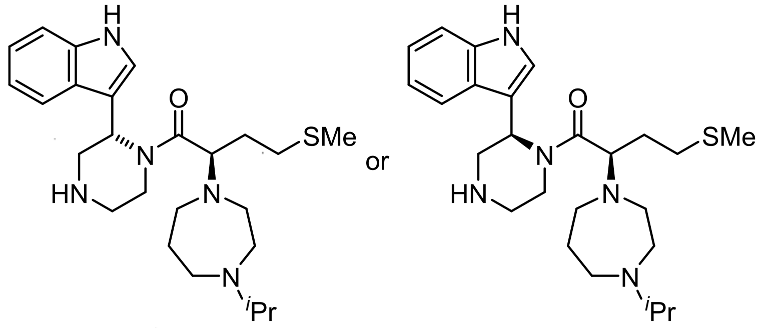

TKB063A (**25A**)

Bruker Compass DataAnalysis 4.2 printed: 1/14/2020 2:36:09 PM by: BDAL@DE 1 of 1

Mass Spectrum SmartFormula Report

Analysis Info

Analysis Name	D:\Data\B\mkurakami\20200114\20200114000005.d	Acquisition Date	1/14/2020 2:38:56 PM
Method	esi_pos_low.m	Operator	BDAL@DE
Sample Name	MKU-1614	Instrument	micrOTOF 000000.00000
Comment	(-)-TKB063 HRMS		

Acquisition Parameter

Source Type	ESI	Ion Polarity	Positive	Set Nebulizer	0.4 Bar
Focus	Not active			Set Dry Heater	200 °C
Scan Begin	50 m/z	Set Capillary	4500 V	Set Dry Gas	4.0 l/min
Scan End	3000 m/z	Set End Plate Offset	-500 V	Set Divert Valve	Source

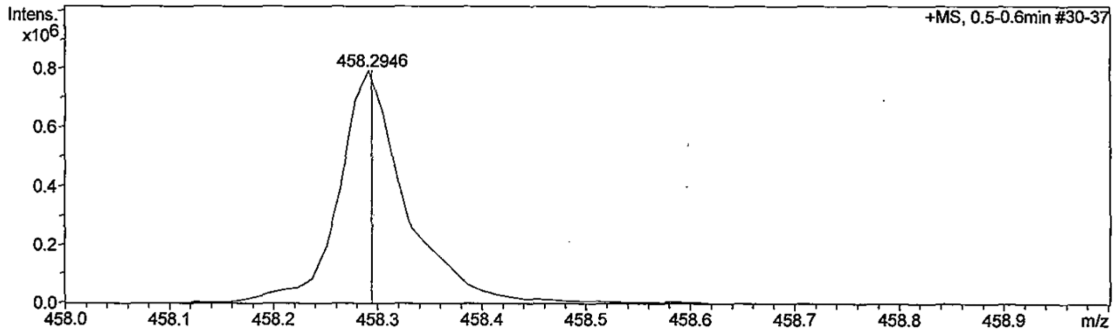

Meas. m/z	#	Ion Formula	m/z	err [ppm]	mSigma	# mSigma	Score	rdb	e⁻ Conf	N-Rule
458.2946	1	C25H40N5OS	458.2948	0.3	4.5	1	100.00	8.5	even	ok

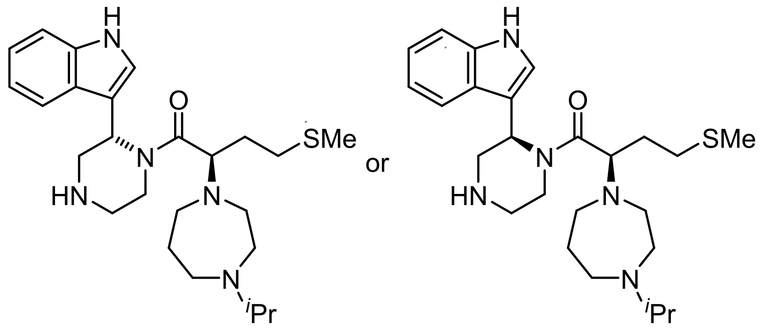

TKB063B (25B)

Bruker Compass DataAnalysis 4.2 printed: 1/14/2020 2:41:07 PM by: BDAL@DE 1 of 1

Mass Spectrum SmartFormula Report

Analysis Info

Analysis Name	D:\Data\B\tonishi\20210115000001.d	Acquisition Date	1/15/2021 12:48:49 PM
Method	esi_pos_wide.m	Operator	BDAL@DE
Sample Name	TON-0233	Instrument	micrOTOF 000000.00000
Comment			

Acquisition Parameter

Source Type	ESI	Ion Polarity	Positive	Set Nebulizer	0.4 Bar
Focus	Not active			Set Dry Heater	200 °C
Scan Begin	50 m/z	Set Capillary	4500 V	Set Dry Gas	4.0 l/min
Scan End	3000 m/z	Set End Plate Offset	-500 V	Set Divert Valve	Source

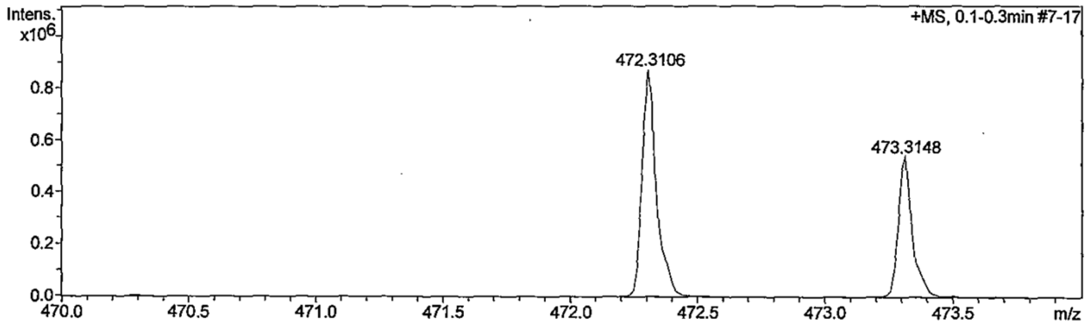

Meas. m/z	#	Ion Formula	m/z	err [ppm]	mSigma	# mSigma	Score	rdb	e⁻ Conf	N-Rule
472.3106	1	C26H42N5OS	472.3105	-0.4	158.3	1	100.00	8.5	even	ok

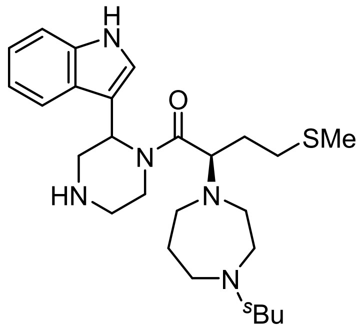

TON03 (**38**)

VI. HPLC charts (compounds 2, 17, 18, 19, 25A, 25B, 26, 27, 29, 36, 37 and 38)

All of the tested compounds (**2**, **17**, **18**, **19**, TKB63A (**25A**), TKB63B (**25B**), **26**, **27**, **29**, TON01 (**36**), TON02 (**37**) and TON03 (**38**)) were >99% purity by HPLC analysis (Figures S1-12).

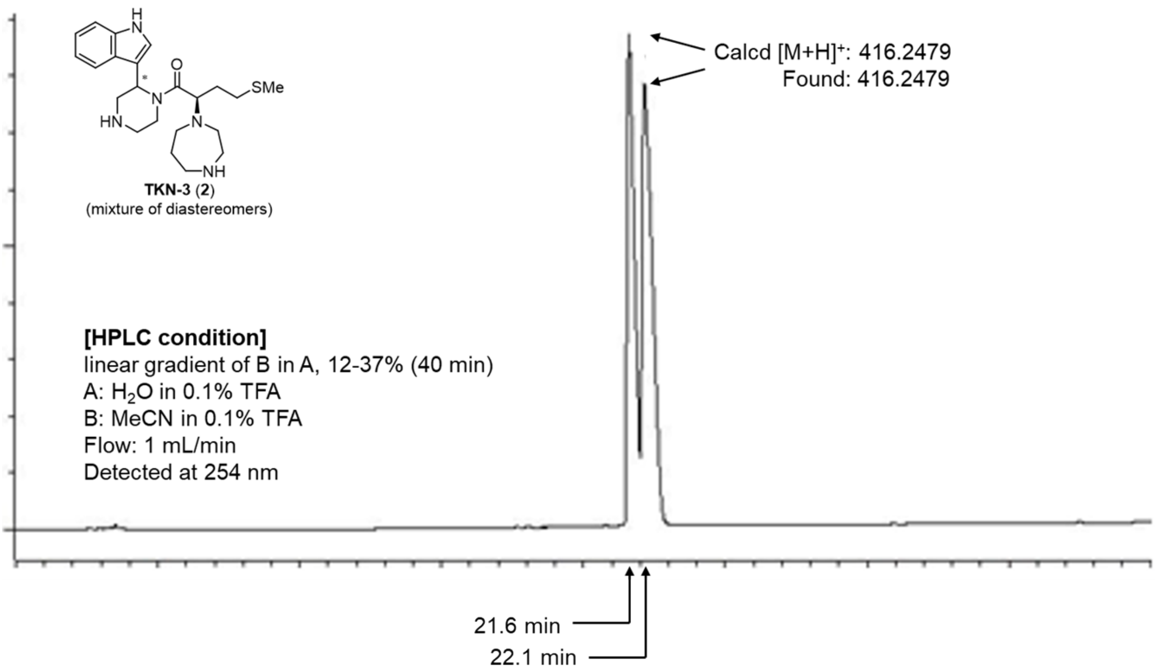

Figure S1. HPLC chart of MKN-3 (**2**).

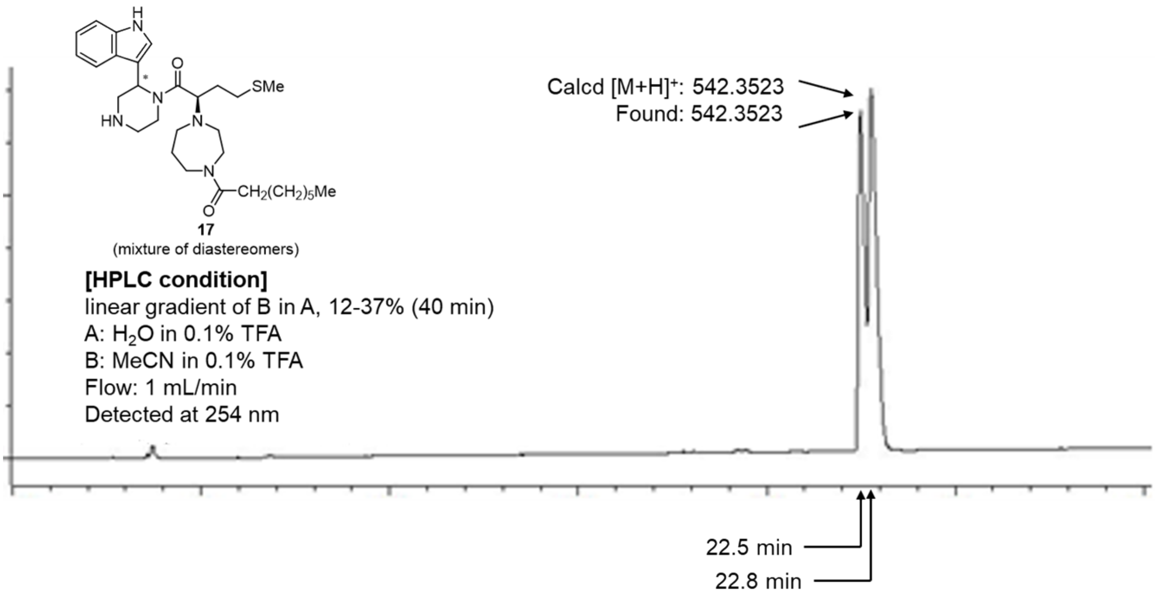

Figure S2. HPLC chart of compound **17**.

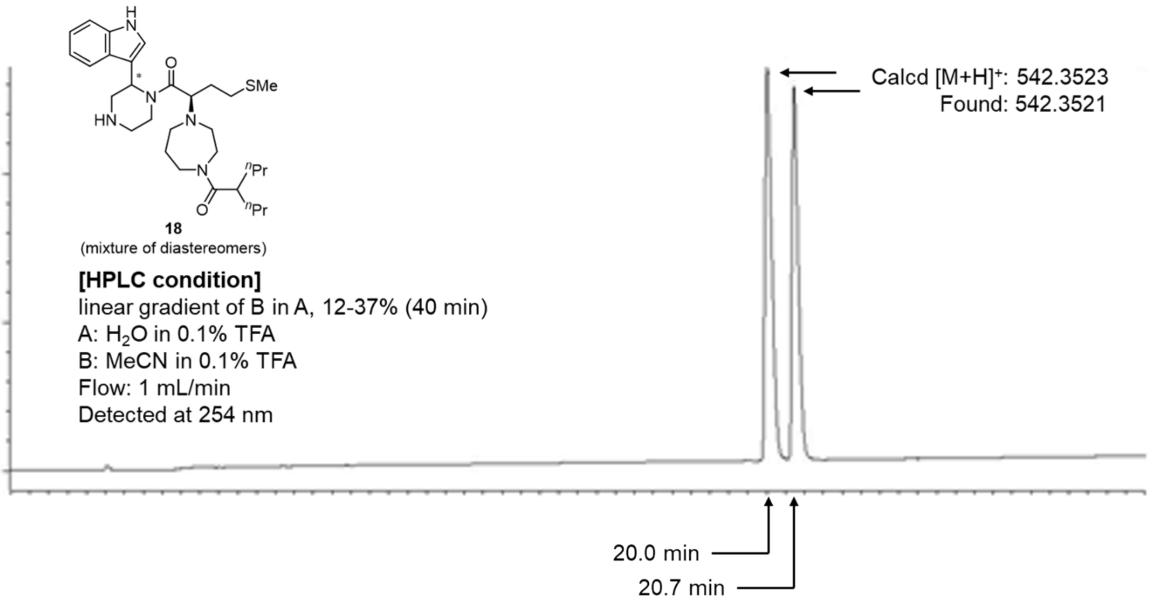

Figure S3. HPLC chart of compound **18**.

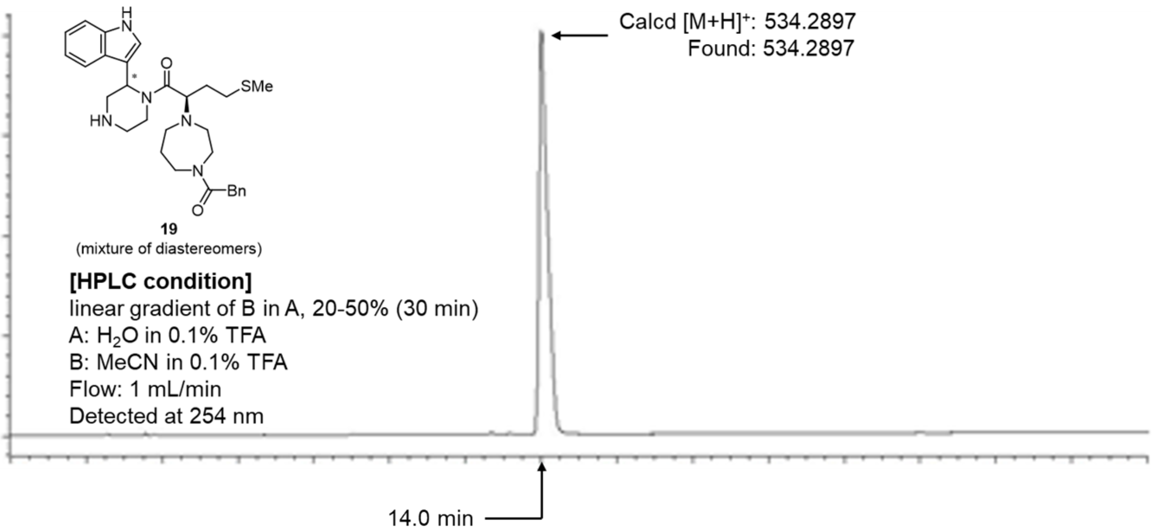

Figure S4. HPLC chart of compound **19**.

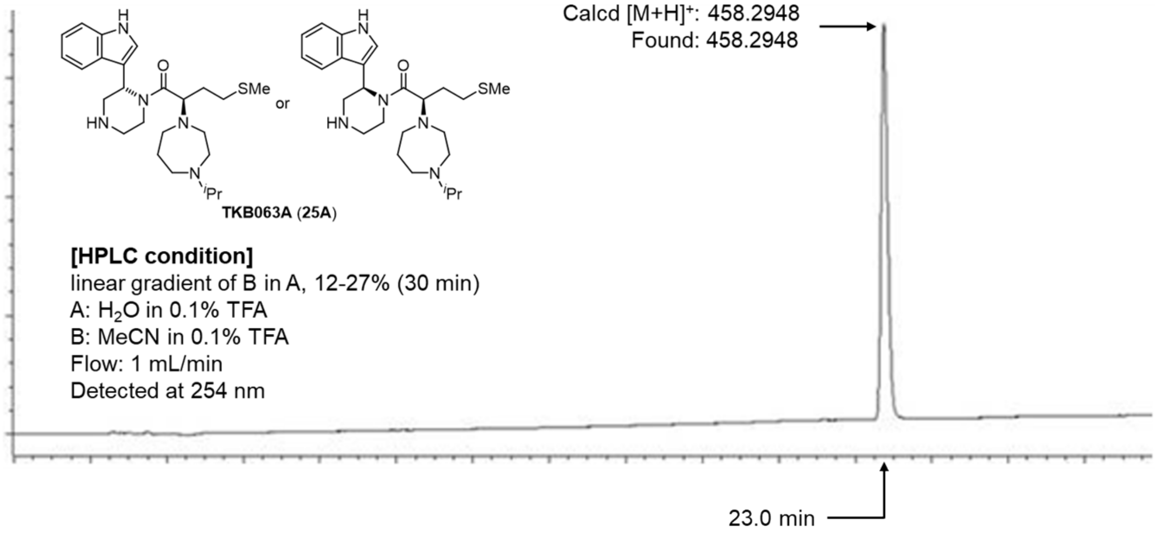

Figure S5. HPLC chart of TKB063A (**25A**).

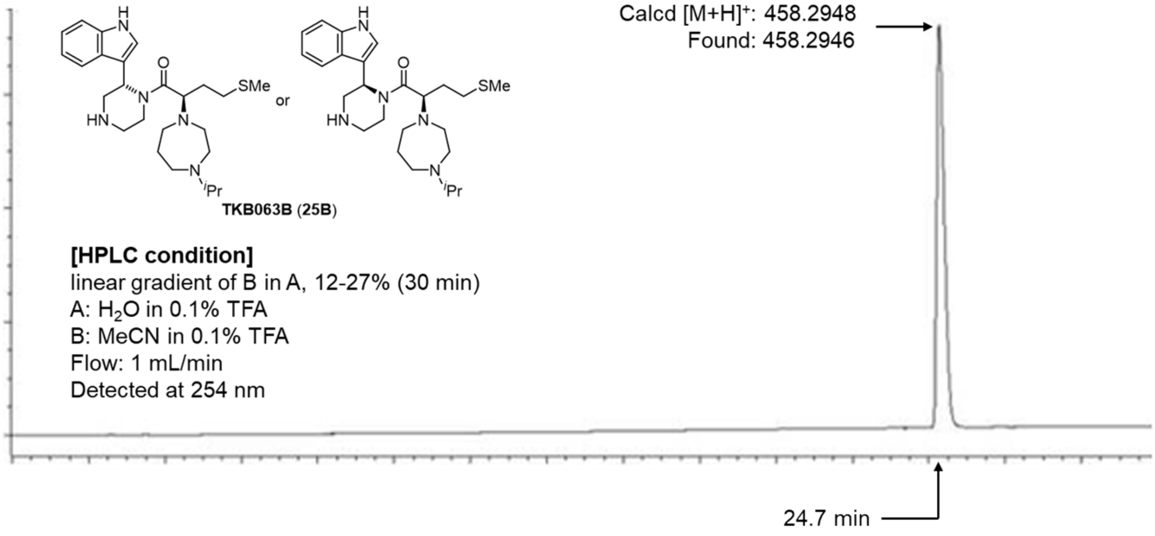

Figure S6. HPLC chart of TKB063B (**25B**).

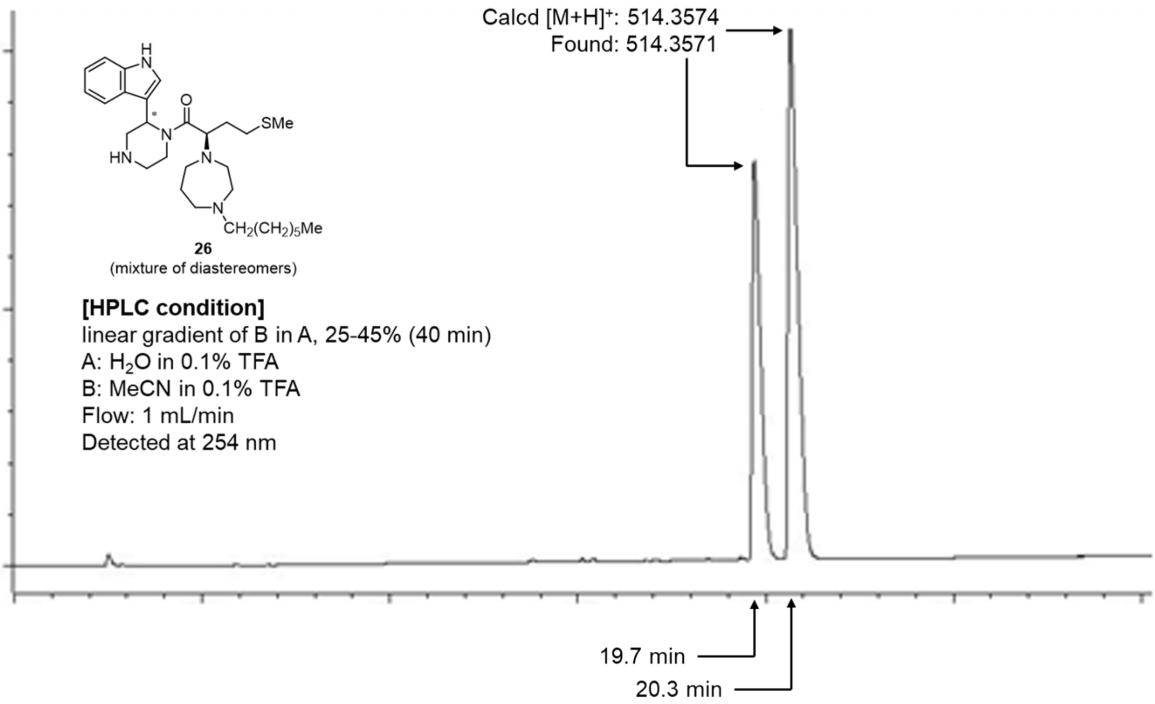

Figure S7. HPLC chart of compound **26**.

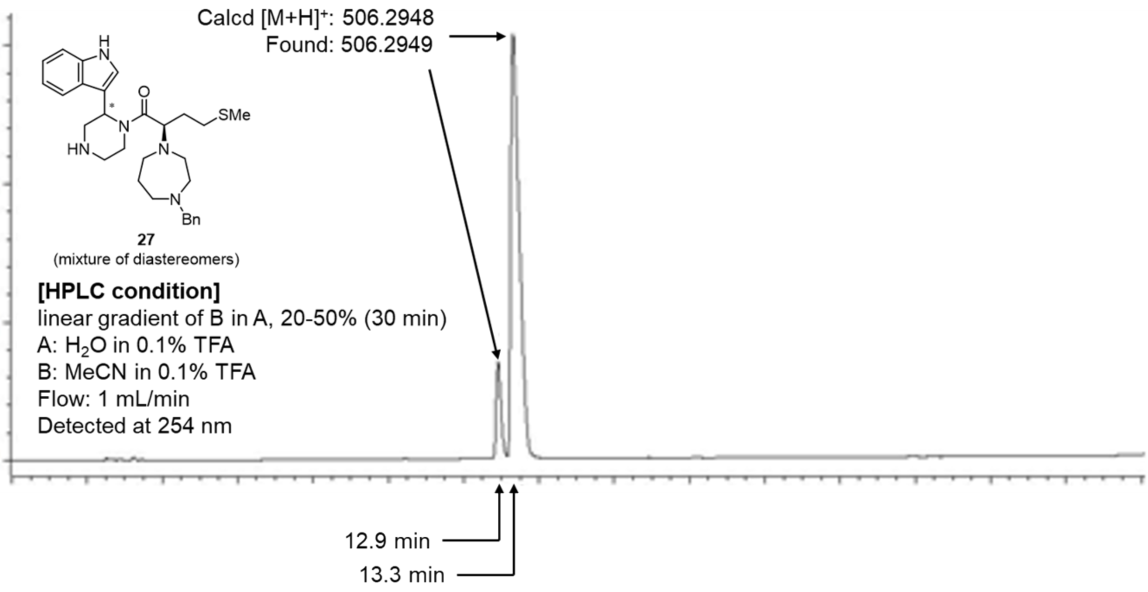

Figure S8. HPLC chart of compound **27**.

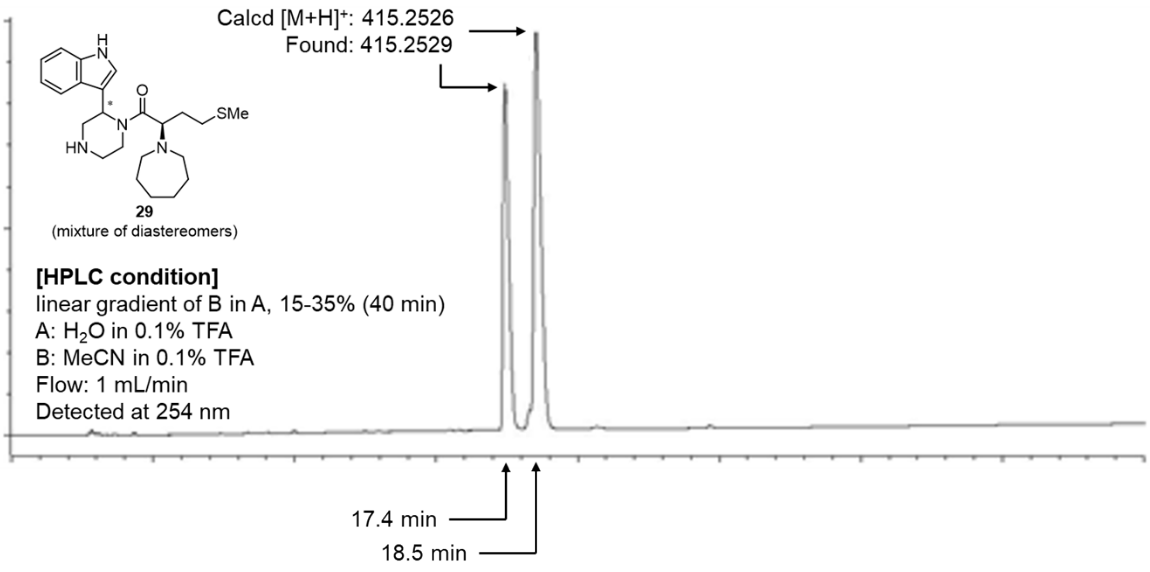

Figure S9. HPLC chart of compound **29**.

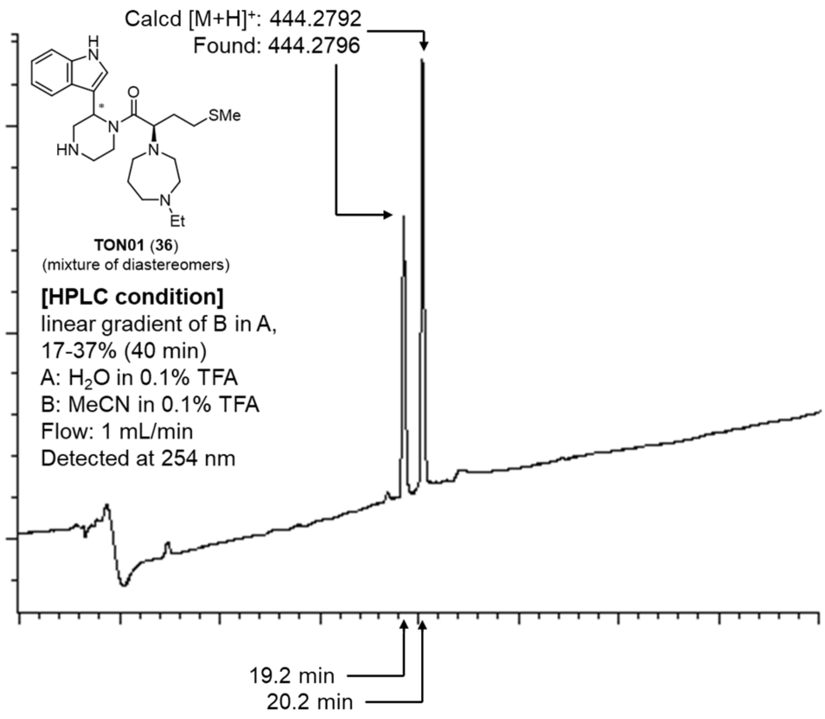

Figure S10. HPLC chart of TON01 (**36**).

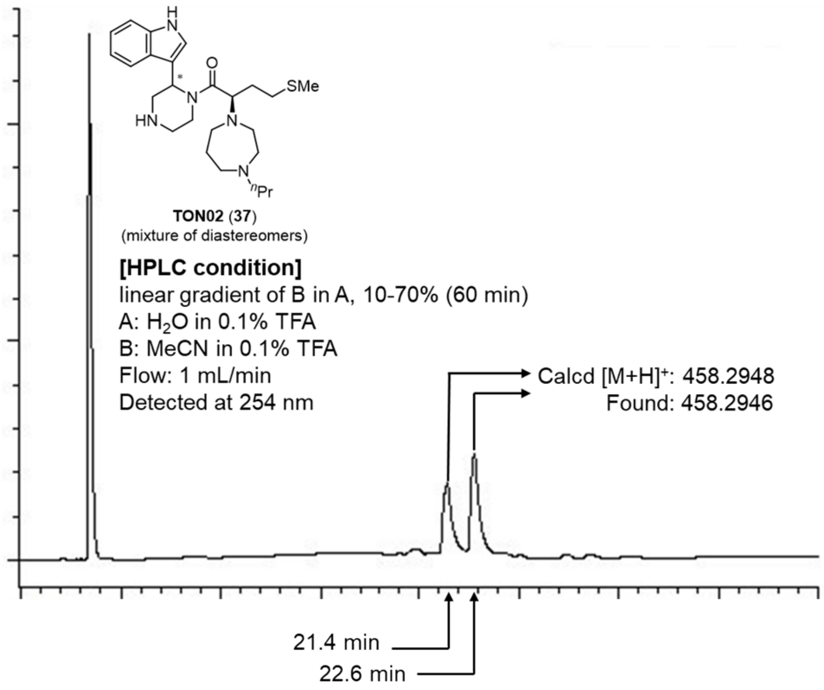

Figure S11. HPLC chart of TON02 (**37**).

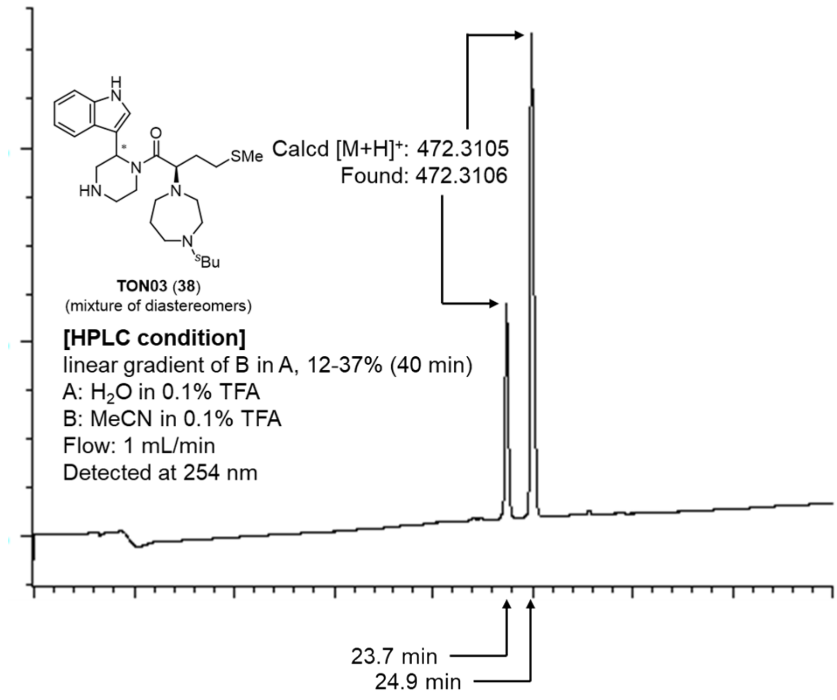

Figure S12. HPLC chart of TON03 (**38**).
